# Rice NAC transcription factor ONAC066 functions as a positive regulator of drought and oxidative stress response

**DOI:** 10.1186/s12870-019-1883-y

**Published:** 2019-06-25

**Authors:** Xi Yuan, Hui Wang, Jiating Cai, Yan Bi, Dayong Li, Fengming Song

**Affiliations:** National Key Laboratory for Rice Biology, Institute of Biotechnology, Zhejiang University, Hangzhou, 310058 China

**Keywords:** Drought tolerance, NAC transcription factor, ONAC066, Oxidative stress tolerance, Rice (*Oryza sativa* L,)

## Abstract

**Background:**

NAC (NAM, ATAF and CUC) transcriptional factors constitute a large family with more than 150 members in rice and several members of this family have been demonstrated to play crucial roles in rice abiotic stress response. In the present study, we report the function of a novel stress-responsive NAC gene, *ONAC066*, in rice drought and oxidative stress tolerance.

**Results:**

ONAC066 was localized in nuclei of cells when transiently expressed in *Nicotiana benthamiana* and is a transcription activator with the binding ability to NAC recognition sequence (NACRS) and AtJUB1 binding site (JBS). Expression of *ONAC066* was significantly induced by PEG, NaCl, H_2_O_2_ and abscisic acid (ABA). Overexpression of *ONAC066* in transgenic rice improved drought and oxidative stress tolerance and increased ABA sensitivity, accompanied with decreased rate of water loss, increased contents of proline and soluble sugars, decreased accumulation of reactive oxygen species (ROS) and upregulated expression of stress-related genes under drought stress condition. By contrast, RNAi-mediated suppression of *ONAC066* attenuated drought and oxidative stress tolerance and decreased ABA sensitivity, accompanied with increased rate of water loss, decreased contents of proline and soluble sugars, elevated accumulation of ROS and downregulated expression of stress-related genes under drought stress condition. Furthermore, yeast one hybrid and chromatin immunoprecipitation-PCR analyses revealed that ONAC066 bound directly to a JBS-like *cis*-elements in *OsDREB2A* promoter and activated the transcription of *OsDREB2A*.

**Conclusion:**

ONAC066 is a nucleus-localized transcription activator that can respond to multiple abiotic stress factors. Functional analyses using overexpression and RNAi-mediated suppression transgenic lines demonstrate that ONAC066 is a positive regulator of drought and oxidative stress tolerance in rice.

**Electronic supplementary material:**

The online version of this article (10.1186/s12870-019-1883-y) contains supplementary material, which is available to authorized users.

## Background

During their lifespan, plants often suffer multiple environmental stresses including drought, salt and extreme temperatures, and thus they have evolved impressive mechanisms at molecular, biochemical, physiological, metabolic and developmental levels to cope with diverse environmental stresses [1–3]. At molecular level, a complicated signaling network is effectively and timely initiated upon perception of external stress signal, and this network ultimately reprograms the expression of a large set of stress-responsive genes via a synergistic action of different types of transcription factors (TFs) in both temporal and spatial manners [1, 3–5]. During the last decade, a number of members in TF families, e.g. NAC (NAM, AFAT and CUC), AP2/ERF, MYB, WRKY, bZIP, homeodomain, bHLH, NF-Y and CAMTA, have been characterized through knockout/knockdown and/or overexpression approaches and demonstrated to play important roles in plant abiotic stress response [6–12].

Among the stress-related TFs identified so far, the plant-specific NAC proteins are characterized by the presence of highly conserved NAC domains at N-terminal, which determine DNA-binding activity, and of variable domains at C-terminal, which are responsible for transcription activity [13]. The NAC recognition sequence (NACRS), containing CATGT and CACG elements, from promoters of various downstream genes, was identified as a core DNA binding site of NAC TFs [14]. NAC TFs constitute a large family with 151 members in rice [15–17]. Recent extensive functional studies have demonstrated that NAC TFs play important roles in regulating biotic and abiotic stress responses in both model and crop plants [8, 12, 18, 19]. Transcriptional profiling analysis revealed that a large portion of the rice ONAC family exhibited overlapping expression patterns in rice under various abiotic and biotic stresses [20, 21]. Functional studies have identified at least 9 rice ONAC genes that play important roles in abiotic stress tolerance and these abiotic stress-related includes *ONAC002* (*SANC1*/*OsNAC9*), *ONAC048* (*SNAC2*/*OsNAC6*), *ONAC009* (*OsNAC5*), *ONAC122* (*OsNAC10*), *ONAC045*, *ONAC058* (*OsNAP*), *ONAC022*, *ONAC095* and *ONAC003* (*SNAC3*) [22–34]. It was found that overexpression of *SNAC1*, *SNAC2* or *ONAC022* significantly enhanced tolerance to dehydration, cold and salt stresses in transgenic rice plants [22, 23], while transgenic rice plants overexpressing the root-specific *OsNAC5*, *OsNAC6*, *OsNAC9*, or *OsNAC10* displayed significant improved drought tolerance [26, 27, 29, 35]. ONAC095 was found to have dual functions in drought and cold stress tolerance [34]. Whereas ONAC095 negatively regulates drought response, it oppositely acts as a positive regulator of cold response in rice [34]. In most of the cases, abscisic acid (ABA)-mediated signaling pathway [22, 23, 25, 29–31], stomatal movement and root system [22, 25, 27–29, 33] were found to be involved in the NAC-mediated improvement of abiotic stress tolerance in transgenic plants. However, SNAC3, which is one of the reported oxidative stress-related NAC TFs, functions as a positive regulator of drought, heat and oxidative stress response through ABA independent pathway [32]. Additionally, transgenic rice lines overexpressing some of the stress-related NAC TFs exhibited significant improvement of abiotic stress tolerance under severe stress conditions without any adverse effect on yield or even with yield increase [22, 26, 27, 29, 30], providing a promising potential for application of these stress-related NAC TFs in improvement of abiotic stress tolerance in crops [36, 37].

Arabidopsis NAC protein JUNGBRUNNEN1 (AtJUB1/ANAC042) was found to function as a central regulator of growth and abiotic stress responses [38–40]. AtJUB1 regulates plant growth via directly repressing the expression of *GA3ox1* and *DWF4* genes encoding key enzymes of gibberellic acid and brassinosteroid biosynthesis and through activating the expression of DELLA genes such as *GA Insensitive* and *RGA-like 1* [41, 42]. Overexpression of *AtJUB1* in Arabidopsis or *MusaNAC042* (banana homologue of *AtJUB1*) in banana resulted in enhanced tolerance to various abiotic stress including drought stress, whereas knockdown of Arabidopsis *AtJUB1* or silencing of tomato *SlJUB1* (tomato homologue of *AtJUB1*) led to decreased tolerance to drought and oxidative stress [38, 39, 43–45]. It was shown that, in regulating the abiotic stress response, the Arabidopsis AtJUB1 and tomato SlJUB1 can directly bind to the promoters of *AtDREB2A*, *SlDREB1* and *SlDREB2*, respectively, which encode AP2-type TFs known to be critical regulators of abiotic stress response [38, 39]. Besides, AtJUB1 acts downstream of the HD-Zip class I TF AtHB13, which is a positive regulator of drought tolerance, establishing a joint drought stress control module between AtHB13 and AtJUB1 [43].

We were interested in the biological functions of rice *ONAC* genes in abiotic and biotic stress response and we previously identified through bioinformatics analysis of publicly available microarray data a number of stress-responsive *ONAC* genes in rice response to biotic and abiotic stresses [20]. Among these stress-responsive *ONAC* genes, *ONAC022* and *ONAC095* were previously shown to play roles in drought, salt and cold stress response [33, 34]. In the present study, we explored the function of ONAC066, the rice homologue of AtJUB1, in regulating stress response using overexpression and RNA interference (RNAi)-mediated suppression transgenic lines. We found that overexpression of *ONAC066* in transgenic rice improved drought and oxidative stress tolerance while RNAi-mediated suppression of *ONAC066* attenuated drought and oxidative stress tolerance. We also showed that ONAC066 bound directly to a JBS-like (JBSL) *cis*-element in *OsDREB2A* promoter and thereby activated transcription of *OsDREB2A*. Our data demonstrate that ONAC066 is a positive regulator of drought and oxidative stress tolerance in rice.

## Results

### *ONAC066* is a putative stress-responsive NAC gene in rice

The *ONAC066* gene (LOC_Os03g56580) contains a complete ORF of 1089 bp, which encodes a polypeptide of 362 amino acid (aa) with a calculated molecular weight of 40.6 kDa and *p*I of 6.4. The ONAC066 protein harbors a single typical conserved NAC domain, spanning 120 aa between 51 aa and 170 aa, at N-terminus, which can be further divided into 5 subdomains (A to E) (Additional file 1: Figure S1A). Phylogenetic tree analysis suggests that ONAC066 belongs to Phylogeny Group IV [16], and is closely related to ONAC096, ONAC140 and ANAC042/AtJUB1 (Additional file 1: Figure S1B), showing 47.7, 37.14 and 34.81% of identity at amino acid level, respectively. Bioinformatics analysis at PlantCARE [46] indicates that the promoter region (1.5 Kb upstream of ATG) of the *ONAC066* gene contains several putative stress-related *cis*- elements, including two ABREs (ABA-responsive element involved in ABA responsiveness, [47]), one CGTCA motif (a *cis*-element involved in JA responsiveness, [48]), one GCC box (a *cis*-element involved in elicitation, wounding and pathogen responsiveness, [49]), two TCA elements (a *cis*-element involved in SA responsiveness, [50, 51]), two TC-rich elements (a *cis*-element involved in defense and stress responsiveness, [52]), and two HSEs (a *cis*-element involved in heat stress responsiveness, [53]) (Additional file 1: Figure S1C). The presence of these stress-responsive *cis*-elements in its promoter suggests that *ONAC066* may be responsive to multiple abiotic stress cues.

### DNA binding and transactivation activities of ONAC066

**Fig. 1:**
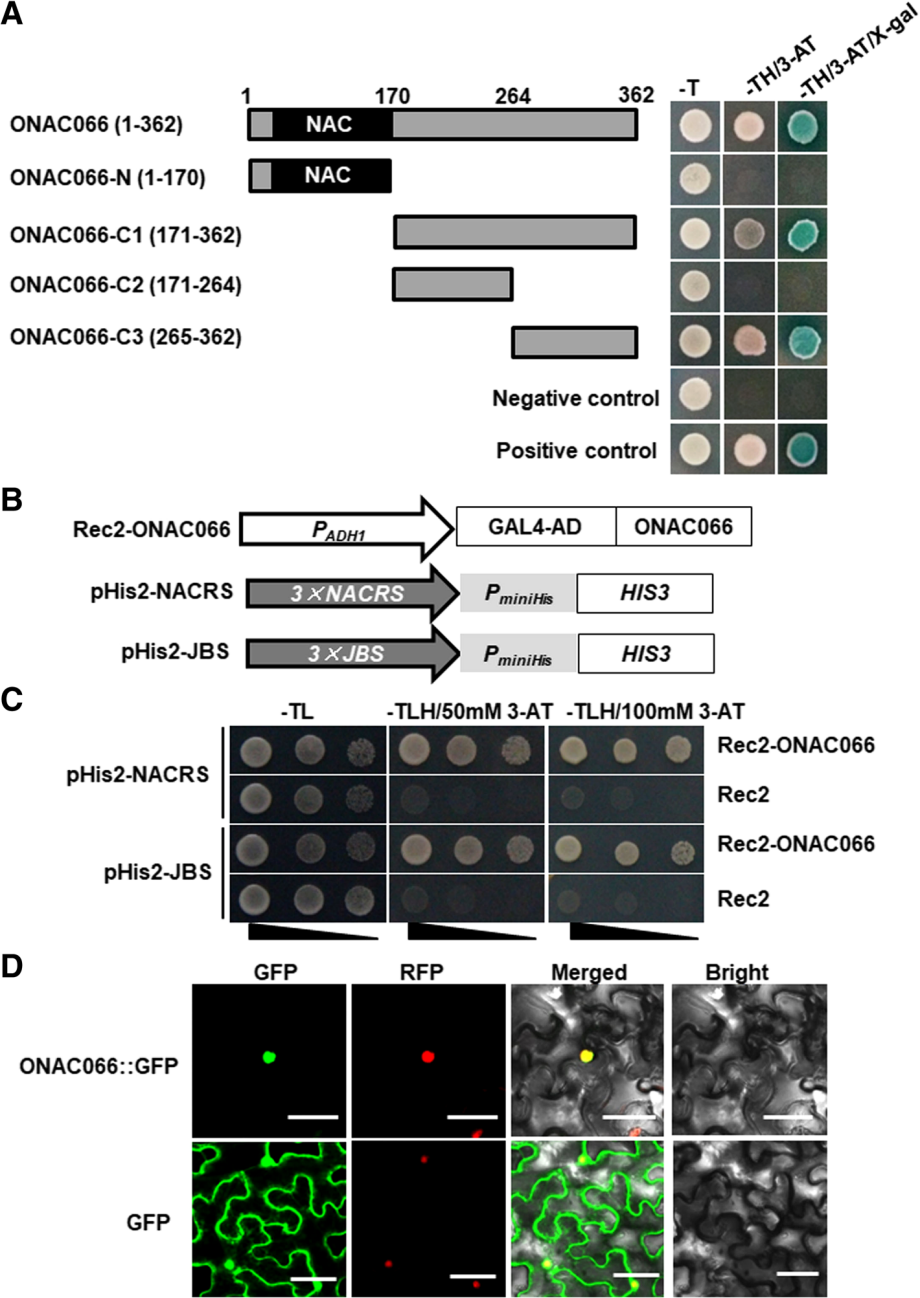
Transactivation activity and subcellular localization of ONAC066. **a** ONAC066 has transactivation activity. Full length and truncated mutants of ONAC066 were fused to GAL4-binding domain and transformed into yeasts. Yeast cells harboring each of the constructs, pGBKT7 empty vector (negative control) or pGBKT7-ONAC022 (positive control) were streaked on medium plates of SD/−Trp, SD/−Trp-His/3-AT and SD/−Trp-His/3-AT/x-α-gal for 3 days at 28 °C. **b** Schematic diagram of different constructs used in ONAC066-NACRS or ONAC066-JBS binding assays. **c** ONAC066 binds to NACRS and JBS in vivo. The Rec2-ONAC066 and Rec2 empty vectors were co-transformed with the reporter constructs pHis-NACRS or pHis-JBS into yeasts and co-transformed yeast cells were streaked by a series of 10-fold dilutions on plates of SD/−Trp-Leu, SD/−Trp-Leu-His, SD/−Trp-Leu-His/50 mM 3-AT and SD/−Trp-Leu-His/100 mM 3-AT. **d** ONAC066 is localized in nucleus. Agrobacteria harboring pCAMBIA1300-ONAC066-GFP or pCAMBIA1300-GFP were infiltrated into leaves of *N. benthamiana* plants expressing a red nucleus marker protein H2B-RFP. Leaf samples were collected at 48 h after agroinfiltration. Microscopic examination was performed under a confocal laser scanning microscope in dark field for green fluorescence (*left*), red fluorescence (*middle left*), white field for cell morphology (*middle right*) and in combination (*right*), respectively. Bar = 10 μm. Experiments in (**a**), (**c**) and (**d**) were repeated for three times with similar results

To determine the TF activity of ONAC066 and, if any, the region in ONAC066 responsible for TF activity, we examined using Y1H assays the transactivation activity of entire ONAC066 and a series of truncated variants including ONAC066-N (1–170 aa), ONAC066-C1 (171–362 aa), ONAC066-C2 (171–264 aa) and ONAC066-C3 (265–362 aa) (Fig. 1a, *left*). Yeast transformants harboring pGBKT7-ONAC066 or one of these variants grew well in SD/−Trp medium (Fig. 1a, *right*). Whereas transformants harboring pGBKT7-ONAC066-N and pGBKT7-ONAC066-C2 failed to grow on SD/−Trp-His/5 mM 3-AT medium, transformants harboring pGBKT7-ONAC066 and variants pGBKT7-ONAC066-C1 and pGBKT7-ONAC066-C3 did grow and show β-galactosidase activity (Fig. 1a, *right*). These results indicate that ONAC066 has a transactivation activity and the region of 265–362 aa at C-terminus is critical for the transactivation activity.

To explore the DNA binding activity of ONAC066, we examined the binding capacity of ONAC066 to NACRS, a canonical NAC core binding sequence [14], and JBS, a novel NAC binding sequence [39], using Y1H assays. For this, tandem repeated 3 × NACRS and 3 × JBS were constructed into pHis2 vector and co-transformed with Rec2-ONAC066 into yeast cells (Fig. 1b). Yeast cells co-transformed with Rec2-ONAC066 and pHis2–3 × NACRS or pHis2–3 × JBS grew normally while yeast cells co-transformed with pGADT7-Rec2 empty vector and pHis2–3 × NACRS or pHis2–3 × JBS failed to grow on SD/−Trp-Leu-His medium with 50 mM or 100 mM 3-AT (Fig. 1c). These results indicate that ONAC066 has DNA binding activity and can bind to both the NAC binding sequences, NACRS and JBS.

### Nucleus localization of ONAC066

The subcellular localization of ONAC066 was examined through transient expression of a GFP-tagged ONAC066 construct in leaves of *Nicotiana benthamiana* plants expressing a red nuclear marker RFP–H2B protein [54]. At 48 h after agroinfiltration, the ONAC066::GFP fusion was microscopically observed to be solely localized in nucleus, which was co-localized with the known nuclear marker RFP–H2B protein (Fig. 1d). In contrast, GFP alone was seen to be ubiquitously distributed throughout the cell without specific localization (Fig. 1d). These results indicate that ONAC066 is a nucleus-localized protein.

### Expression patterns of *ONAC066* upon abiotic stress treatment

**Fig. 2:**
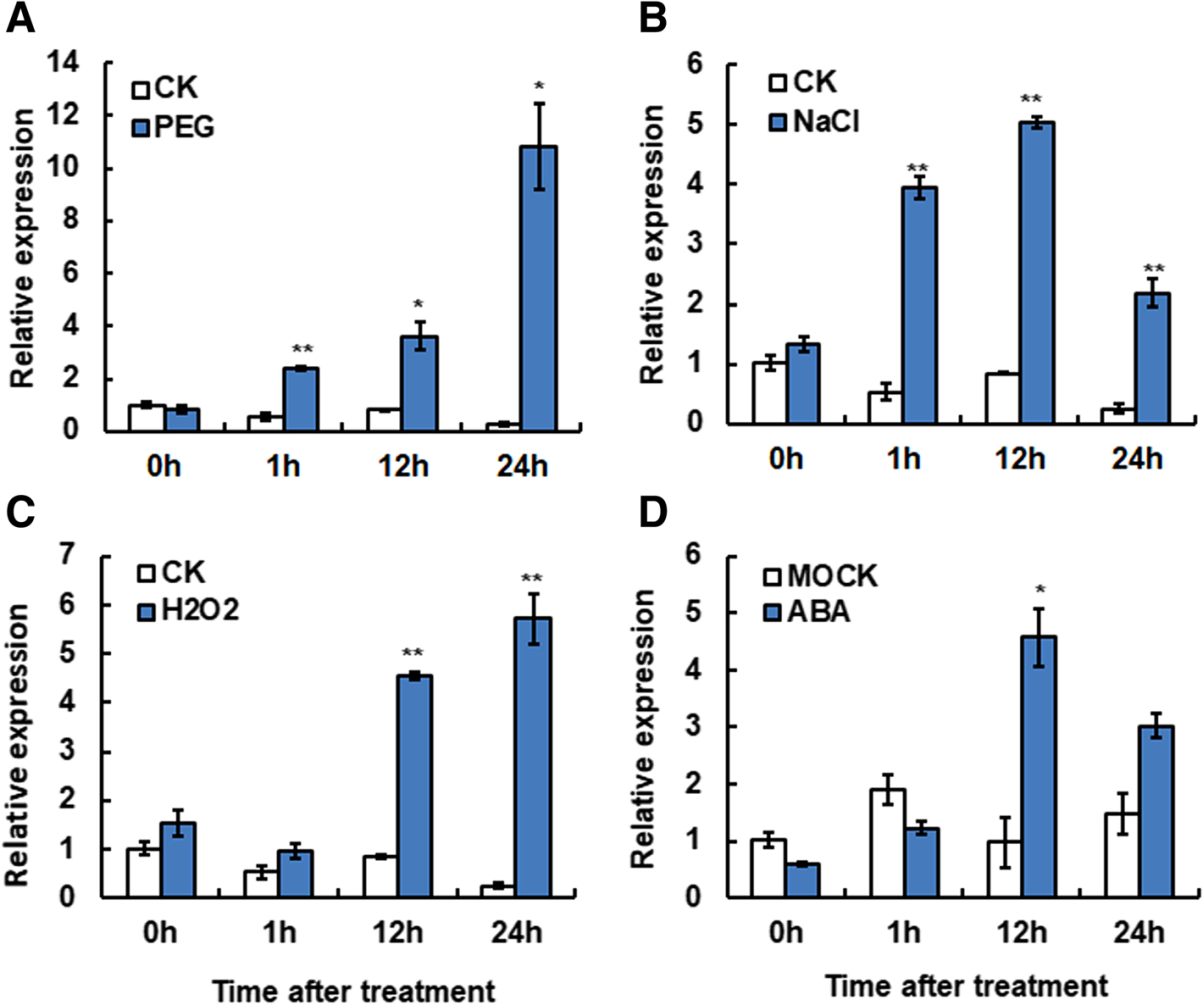
Stress-inducible expression of *ONAC066*. Three-week-old seedlings were exposed to (**a**) 20% PEG6000, **b** 100 mM NaCl, **c** 100 mM H_2_O_2_ or (**d**) 50 μM ABA. Samples were collected at different time intervals and the transcript level of *ONAC066* was quantified by qRT-PCR. Relative expression level of *ONAC066* was normalized to the internal control of *18 s-rRNA* gene. Data presented are the means ± SD from three independent experiments

*ONAC066* is expressed in all tissues, especially in leaves and seeds, as examined using public microarray data at NCBI (http://www.ncbi.nlm.nih.gov/geo) under accession number GSE6893 (Additional file 2: Figure S2). We examined by quantitative real time-PCR (qRT-PCR) the responsiveness of *ONAC066* to abiotic stresses and exogenous ABA. The expression level of *ONAC066* in PEG6000-treated plants started to increase at 1 h after treatment (hpt) and increased gradually over a period of 24 h, with a peak at 24 hpt, showing 35-fold of increase over that in control plants (Fig. 2a). High levels of *ONAC066* expression were observed in NaCl-treated plants at 1 and 12 hpt, showing 6.8- and 5.3-fold of increase over that in control plants (Fig. 2b). In H_2_O_2_-treated plants, significant induction of *ONAC066* expression was detected at 12 and 24 hpt, leading to 4.8- and 18-fold of increased over those in control plants (Fig. 2c). In ABA-treated plants, the expression level of *ONAC066* showed significant increase only at 12 hpt, giving a 3.6-fold increase over that in control plants (Fig. 2d). These results indicate that *ONAC066* is an abiotic stress- and ABA-responsive rice ONAC gene.

### Generation of ONAC066-OE and ONAC066-RNAi transgenic lines

To investigate the function of *ONAC066*, we generated ONAC066-OE and ONAC066-RNAi transgenic rice lines in *japonica* rice cv. Zhonghua11 (ZH11) background (Additional file 3: Figure S3A). qRT-PCR revealed that the transcript levels of *ONAC066* in ONAC066-OE lines OE11 and OE12 showed 57.1- and 79.9-fold higher than that in wild type (WT) plants, while the levels in ONAC066-RNAi lines Ri1 and Ri21 were estimated to be 34 and 39% of the level in WT plants (Additional file 3: Figure S3B). The ONAC066-GFP fusion in ONAC066-OE lines OE11 and OE12 plants was detected with GFP antibody, showing a band of ~ 67.8 kD that matched the molecular weight of the fusion (Additional file 3: Figure S3C). No significant defect in morphological, growth and developmental characters was observed in ONAC066-OE and ONAC066-RNAi plants.

### Overexpression of *ONAC066* improved while suppression of *ONAC066* weakened drought tolerance

**Fig. 3:**
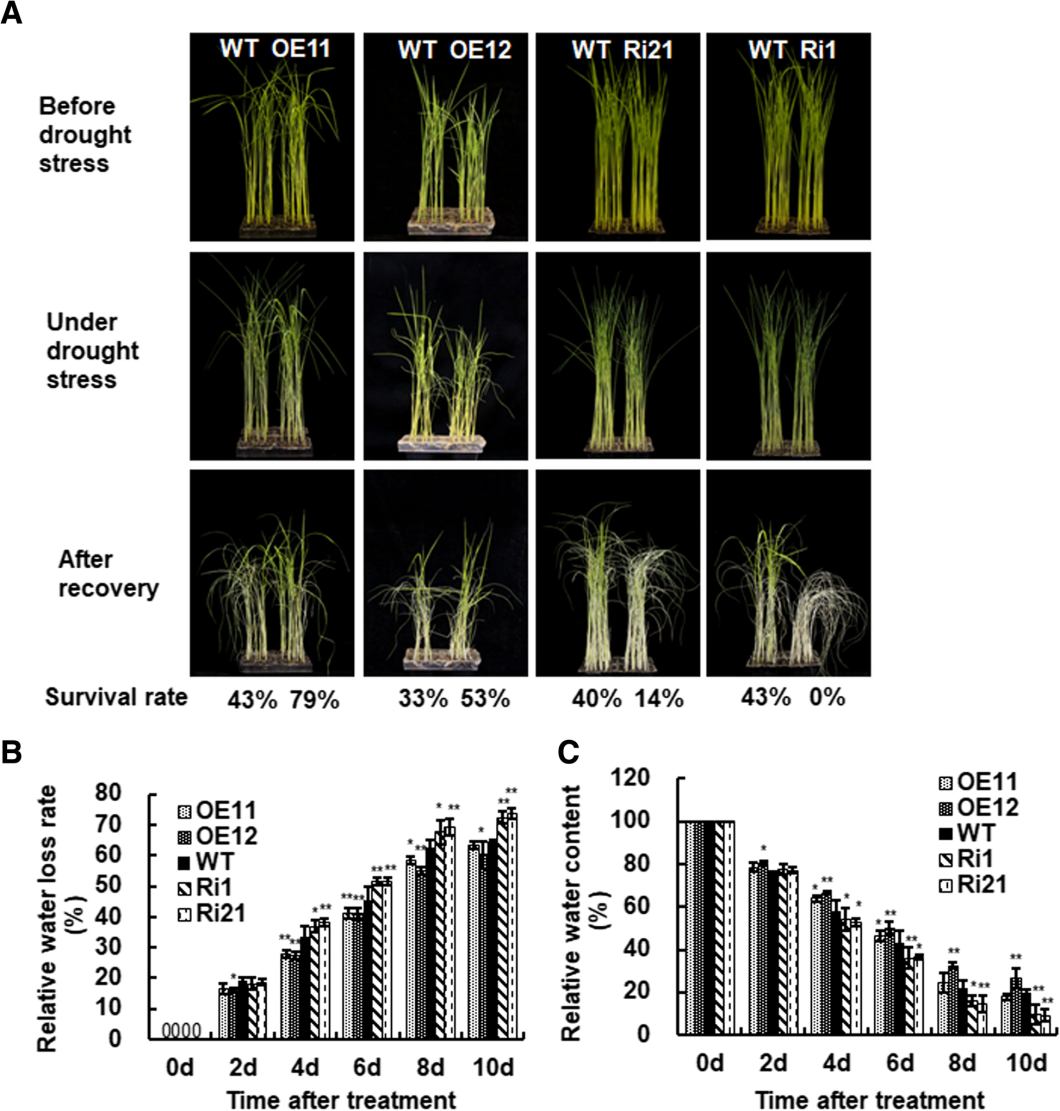
ONAC066-OE plants increased while ONAC066-RNAi plants decreased drought stress tolerance. **a** Growth phenotype of 3-week-old transgenic ONAC066-OE, ONAC066-RNAi and WT plants at different stages during drought stress experiments. Survival rates for each line after recovery were measured at 2 days after re-watering. **b** Relative water loss rate and (**c**) relative water content in drought-treated plants. Parameters for water status in 3-week-old ONAC066-OE, ONAC066-RNAi and WT plants after drought stress treatment were measured at indicated time points over a period of 10 d after treatment. Experiments in (**a**) were repeated for three times with similar results. Data presented in (**b**) and (**c**) are the means ± SD from three independent experiments and difference between ONAC066-OE/ONAC066-RNAi and WT plants are indicated by asterisks (*p < 0.05 or **p < 0.01) according to Student *t* test

We first explored whether *ONAC066* played a role in drought tolerance by phenotyping ONAC066-OE and ONAC066-RNAi plants under drought condition and comparing with WT plants. Under normally watered condition, the growth phenotype of ONAC066-OE and ONAC066-RNAi plants were indistinguishable from WT plants (Fig. 3a, *upper panel*). At 7 days after drought treatment by water withholding, the ONAC066-RNAi plants showed earlier and severer drought symptom, represented by rolled leaves and wilted plants, while the ONAC066-OE plants displayed relatively later and slighter drought symptom, as compared with the WT plants (Fig. 3a, *middle panel*). At 2 days after re-watering, 79 and 53% of ONAC066-OE lines OE11 and OE12 plants recovered and survived, showing 36 and 20% higher over those in WT plant (43 and 33%) (Fig. 3a, *lower panel*). By contrast, only 14% of the ONAC066-RNAi line Ri21 plants and none of the Ri1 plants recovered and survived, showing 26 and 43% lower over those in WT plant (40 and 43%) (Fig. 3a, *lower panel*). These results indicated that overexpression of *ONAC066* improved while suppression of *ONAC066* weakened drought tolerance in transgenic rice.

To further confirm the involvement of ONAC066 in drought tolerance, we compared water status, as reflected by water loss rate (WLR) and relative water content (RWC), in ONAC066-OE and ONAC066-RNAi plants with those in WT plants after drought stress treatment. As compared to those in WT plants, WLR in drought stress-treated ONAC066-OE plants was significantly reduced by 4–8%, while WLR in drought stress-treated ONAC066-RNAi plants was increased by 5–9% at 4, 6, 8 and 10 d after treatment, respectively (Fig. 3b). As consequences, the drought stress-treated ONAC066-OE plants had significantly higher RWC, showing 8–12% of increase, while the drought stress-treated ONAC066-RNAi line Ri1 plants showed lower RWC, giving 5–10% of decrease, as compared to those in drought stress-treated WT plants, at 4, 6, 8 and 10 d after treatment (Fig. 3c). These results further confirm that ONAC066 plays a significant role in rice drought stress tolerance.

### Overexpression of *ONAC066* enhanced while suppression of *ONAC066* attenuated oxidative stress tolerance

**Fig. 4:**
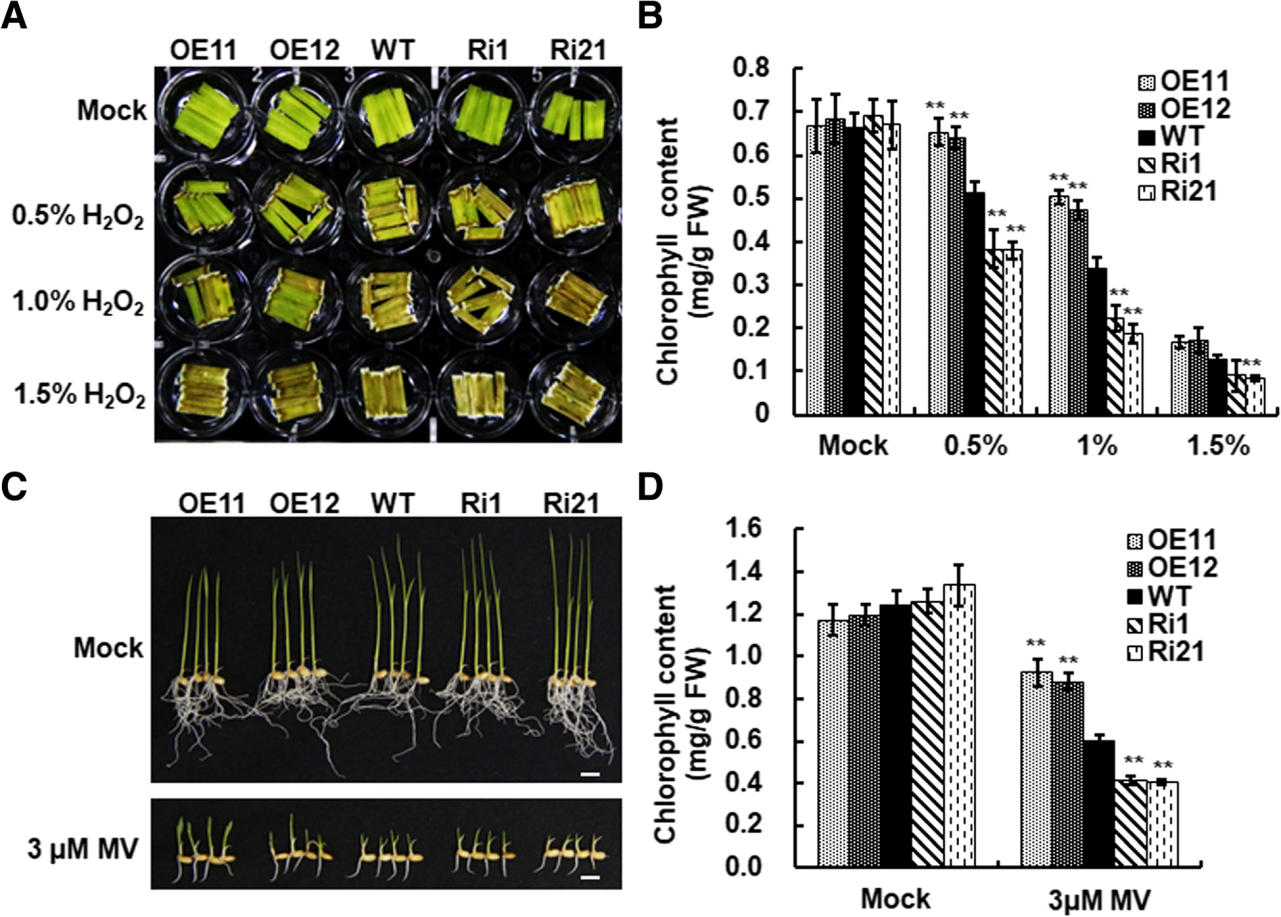
ONAC066-OE plants increased while ONAC066-RNAi plants decreased oxidative stress tolerance. **a** Sensitivity of ONAC066-OE, ONAC066-RNAi and WT plants to exogenous H_2_O_2_. Leaf fragments were collected from 3-week-old ONAC066-OE, ONAC066-RNAi and WT plants grown under normal condition and were exposed to H_2_O_2_ at different concentrations for 48 h. **b** Chlorophyll contents in leaf fragments after H_2_O_2_ treatment in (**a**). **c** Sensitivity of ONAC066-OE, ONAC066-RNAi and WT plants to MV. Germinated seeds of ONAC066-OE, ONAC066-RNAi and WT lines were planted on 1/2 MS medium with or without 3 μM MV and allowed for growth for 10 days. **d** Chlorophyll contents in leaves of ONAC066-OE, ONAC066-RNAi and WT plants after MV treatment in (**c**). Experiments in (**a**) and (**c**) were repeated for three times with similar results. Data presented in (**b**) and (**d**) are the means ± SD from three independent experiments and difference between ONAC066-OE/ONAC066-RNAi and WT plants are indicated by asterisks (**p < 0.01) according to Student *t* test. FW: fresh weight

It was reported that overexpression of *AtJUB1*, an Arabidopsis homologue of ONAC066 (Additional file 1: Figure S1B), conferred tolerance to exogenous H_2_O_2_ [39]. We thus examined whether *ONAC066* played a role in rice oxidative stress tolerance. Without H_2_O_2_ treatment, no significant phenotype appeared on leaf discs from 3-week-old ONAC066-OE, ONAC066-RNAi and WT plants during a period of 48 h in the H_2_O_2_ tolerance assays (Fig. 4a). At 48 h after treatment in H_2_O_2_ solution at concentrations of 0.5, 1.0 and 1.5%, yellowing and chlorosis symptoms were observed in leaf discs from ONAC066-OE, ONAC066-RNAi and WT plants (Fig. 4a); however, yellowing and chlorosis symptoms in leaf discs from ONAC066-OE plants were less severe while these symptoms in leaf discs from ONAC066-RNAi plants were much evident, as compared with those in WT plants (Fig. 4a). These observations were confirmed by measuring chlorophyll contents in leaf discs from ONAC066-OE, ONAC066-RNAi and WT plants after H_2_O_2_ treatments (Fig. 4b). Without H_2_O_2_ treatment, chlorophyll contents in leaf discs from ONAC066-OE and ONAC066-RNAi plants were comparable to that in WT plant (Fig. 4b). However, chlorophyll contents in leaf discs from ONAC066-RNAi lines Ri1 and Ri21 plants were dramatically decreased, leading to 25–26%, 34–45% and 29–35% of reduction, as compare with those in WT plants, at 48 h after treatment in 0.5, 1.0% or 1.5% H_2_O_2_ solution, respectively (Fig. 4b). By contrast, chlorophyll contents in leaf discs from ONAC066-OE lines OE11 and OE12 plants were significantly higher, showing 25–27% and 40–49% of increase, as compare with those in WT plants, at 48 h after treatment in 0.5% or 1.0% H_2_O_2_ solution, respectively (Fig. 4b). These results indicate that overexpression of *ONAC066* increased while suppression of *ONAC066* attenuated the tolerance to exogenous H_2_O_2_.

We further explored whether modification of *ONAC066* affected the tolerance of transgenic rice plants to methyl viologen (MV) (a compound generating reactive oxygen species including superoxide anion), by comparing growth phenotype of and chlorophyll content in ONAC066-OE and ONAC066-RNAi seedlings grown on 1/2 Murashige and Skoog (MS) medium supplemented with MV to those of WT seedlings. In 1/2 MS medium without supplementation of MV, slight suppression or promotion in growth of ONAC066-OE and ONAC066-RNAi seedlings, respectively, were observed, as compared with that of WT seedlings (Fig. 4c). As compared with growth of the seedlings grown MV-free 1/2 MS, growth of ONAC066-OE, ONAC066-RNAi and WT seedlings grown on 1/2 MS supplemented with 3 μM MV were significantly suppressed (Fig. 4c). On medium with MV, ONAC066-OE seedlings showed less growth suppression while ONAC066-RNAi seedlings exhibited severer growth suppression, as compared to WT seedlings (Fig. 4c). Similarly, slight increase or decrease in chlorophyll contents in ONAC066-OE and ONAC066-RNAi seedlings, respectively, were observed, as compared with that in WT seedlings, when grown in 1/2MS medium without supplementation of MV (Fig. 4d). However, when grown on medium with MV, chlorophyll contents in ONAC066-OE, ONAC066-RNAi and WT seedlings were significantly decreased, as compared with that in seedlings grown in MV-free medium (Fig. 4d). Chlorophyll contents in ONAC066-OE lines OE11 and OE12 seedlings showed 47–55% higher while those in ONAC066-RNAi lines Ri1 and Ri21 seedlings exhibited 31–32% lower than that in WT seedlings (Fig. 4d). These results indicate that overexpression of *ONAC066* increased while suppression of *ONAC066* attenuated the tolerance to MV.

### Overexpression of *ONAC066* increased while suppression of *ONAC066* decreased ABA sensitivity

**Fig. 5:**
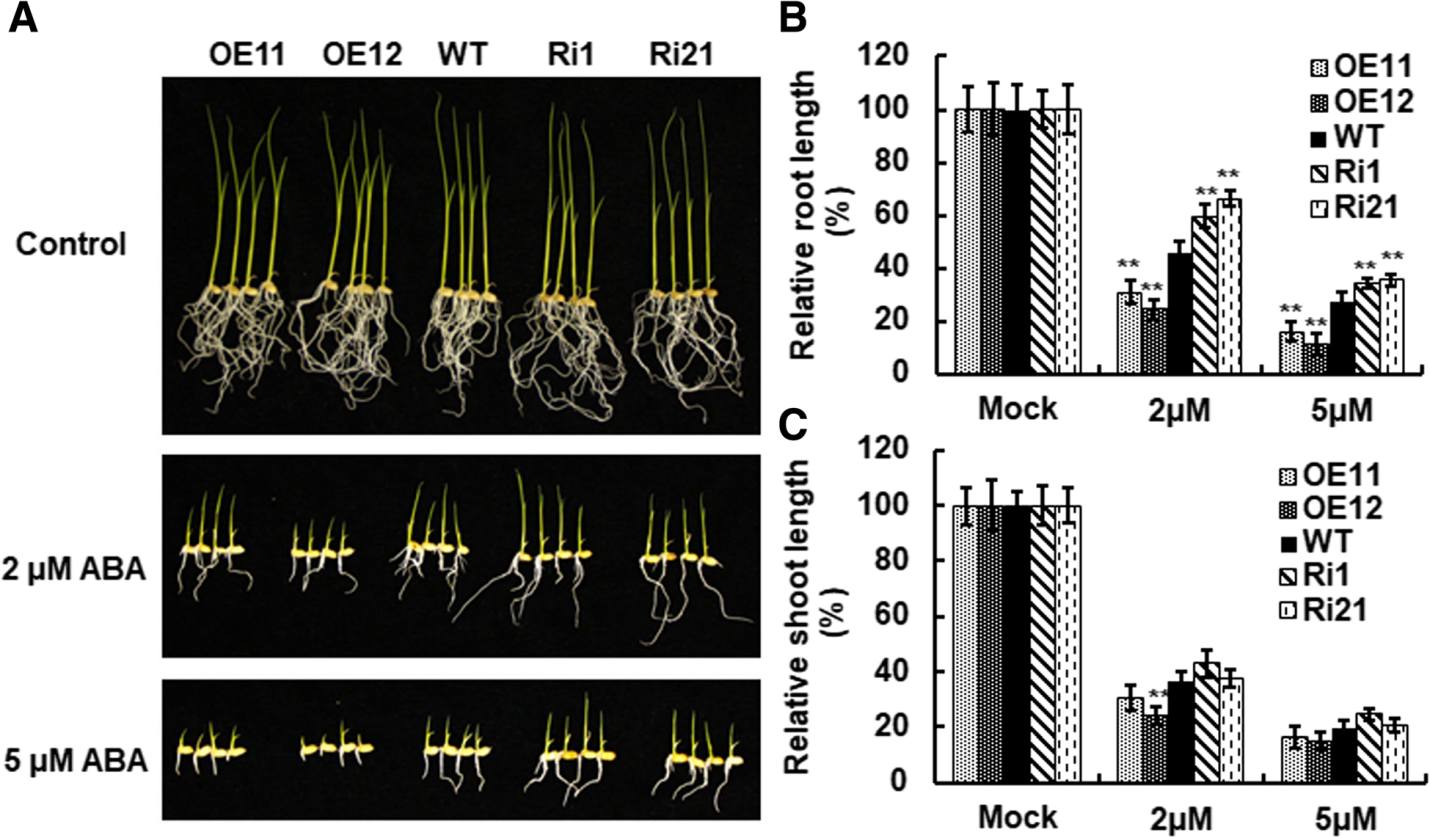
ONAC066-OE plants increased while ONAC066-RNAi plants decreased ABA sensitivity. **a** Growth performance of ONAC066-OE, ONAC066-RNAi and WT seedlings grown on 1/2 MS medium with or without ABA. **b** Relative root length and (**c**) relative sheath length of ONAC066-OE, ONAC066-RNAi and WT seedlings grown on 1/2 MS with or without ABA at 10 days after germination. Experiments in (**a**) were repeated for three times with similar results. Data presented in (**b**) and (**c**) are the means ± SD from three independent experiments and difference between ONAC066-OE/ONAC066-RNAi and WT plants are indicated by asterisks (**p < 0.01) according to Student *t* test

The fact that expression of *ONAC066* was induced by ABA led us to explore whether altered expression of ONAC066 affected ABA sensitivity in ONAC066-OE and ONAC066-RNAi plants. We examined the ABA sensitivity of ONAC066-OE and ONAC066-RNAi seedlings and compared with WT seedlings by analyzing seedling growth in the presence of ABA. Growth of ONAC066-OE, ONAC066-RNAi seedlings was indistinguishable from WT seedlings in the absence of ABA, but their growth was suppressed when grown on 1/2 MS supplemented with ABA (Fig. 5a). However, growth of ONAC066-OE seedlings was severely suppressed while ONAC066-RNAi seedlings was slightly suppressed, as compared with that of WT seedlings, when grown on 1/2 MS supplemented with 2 μM or 5 μM ABA (Fig. 5a). Root length of ONAC066-OE lines OE11 and OE12 seedlings was significantly shorted, resulting in 32–41% and 47–59% of reduction, while root length of ONAC066-RNAi lines Ri1 and Ri21 seedlings were longer, leading to 25–31% and 31–45%, respectively, than those in WT seedlings, when grown on 1/2 MS supplemented with 2 μM or 5 μM ABA (Fig. 5b). By contrast, on ABA-containing 1/2 MS, shoot length of ONAC066-OE and ONAC066-RNAi seedlings were comparable to that of WT seedlings, except for a significant reduction in shoot length of ONAC066-OE line OE12 seedlings on 1/2 MS containing 2 μM ABA (Fig. 5c). These results indicate that overexpression of *ONAC066* increased while suppression of *ONAC066* decreased ABA sensitivity.

### Altered expression of *ONAC066* affected the accumulation of stress-related metabolites during drought stress response

**Fig. 6:**
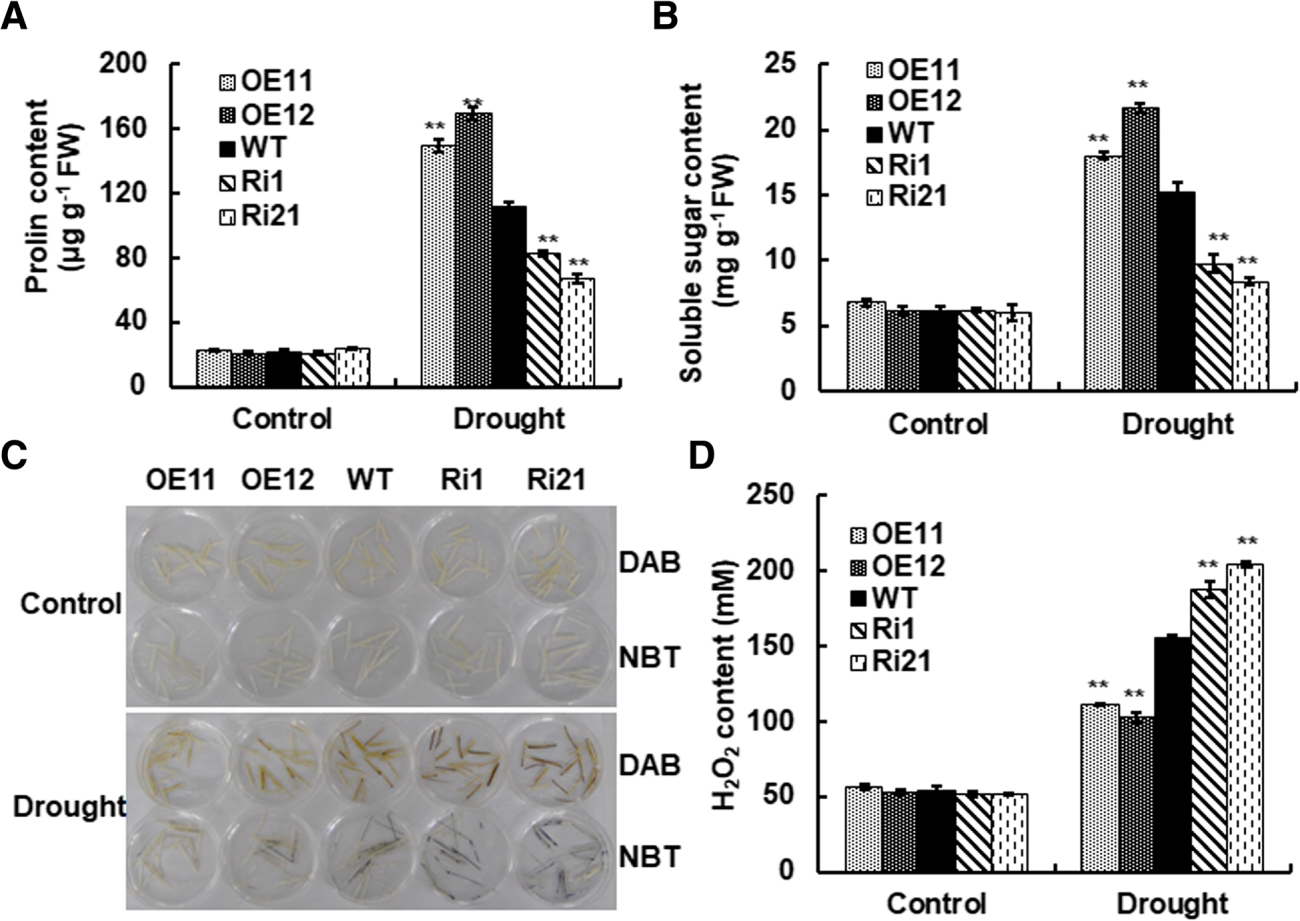
Drought-induced accumulation of proline, soluble and ROS in ONAC066-OE and ONAC066-RNAi plants. **a** Proline content and (**b**) soluble sugar content. **c** In situ detection of H_2_O_2_ and superoxide anion in leaves by DAB and NBT staining, respectively. **d** H_2_O_2_ levels in leaves. Leaf samples were collected from 3-week-old ONAC066-OE, ONAC066-RNAi and WT plants grown under normally watered and drought-stressed (at 5 days after water withholding) conditions and were subjected to physiological measurements. Experiments in (**c**) were repeated for three times with similar results. Data presented in (**a**), (**b**) and (**d**) are the means ± SD from three independent experiments and significant difference between ONAC066-OE/ONAC066-RNAi and WT plants under the same treatment are indicated by asterisks (**p < 0.01 and *p < 0.05) according to Student *t* test. FW: fresh weight

To explore the possible physiological basis of ONAC066-mediated stress response, we analyzed and compared the changes in some stress-related metabolites such as proline and soluble sugars in ONAC066-OE and ONAC066-RNAi plants with those in WT plants under normal watering and water withholding drought conditions. Under normally watered condition, contents of proline and soluble sugars in both ONAC066-OE and ONAC066-RNAi plants were comparable to those in WT plants (Fig. 6a and b). At 5 days after water withholding drought treatment, contents of proline and soluble sugars were increased in ONAC066-OE, ONAC066-RNAi and WT plants as compared with those in their corresponding control plants grown under normal watering condition (Fig. 6a and b). The ONAC066-OE lines OE11 and OE12 plants showed higher contents of proline and soluble sugars, leading to 34–52% and 18–42% of increase, respectively, while the ONAC066-RNAi lines Ri1 and Ri21 plants exhibited lower contents of proline and soluble sugars, giving 26–40% and 37–45% of decrease, respectively, as compared with those in WT plants, under water withholding drought condition (Fig. 6a and b). These results indicate that overexpression of *ONAC066* stimulated while suppression of *ONAC066* decreased the accumulation of stress-related metabolites such as proline and soluble sugars during drought stress response.

ROS is often involved in abiotic stress response by causing oxidative damage. We thus analyzed and compared the accumulation of ROS in ONAC066-OE and ONAC066-RNAi plants with those in WT plants under normally watering and water withholding drought conditions to explore the involvement of ROS in ONAC066-mediated drought stress response. Qualitative staining by 3,3′-diaminobenzidine (DAB) and nitroblue tetrazolium (NBT) and quantitative measurement indicated that staining of H_2_O_2_ and superoxide anion and accumulation of H_2_O_2_ in leaf tissues of ONAC066-OE and ONAC066-RNAi plants were indistinguishable from those in WT plants grown under normally watering condition (Fig. 6c and d). Staining of H_2_O_2_ and superoxide anion was much evident and the accumulation levels of H_2_O_2_ were increased in leaf tissues of ONAC066-OE, ONAC066-RNAi and WT plants than those in their corresponding control plants grown under normally watering condition (Fig. 6c and d). At 5 days after water withholding drought treatment, staining of H_2_O_2_ and superoxide anion in leaf tissues of ONAC066-OE plants was less evident while the staining in leaf tissues of ONAC066-RNAi plants was much obvious, as compared with those in WT plants (Fig. 6c). Accordingly, the ONAC066-OE lines OE11 and OE12 plants showed higher H_2_O_2_ accumulation level, leading to 28–34% of reduction, while the ONAC066-RNAi lines Ri1 and Ri21 plants exhibited lower contents of proline and soluble sugars, giving 21–31% of increase, as compared with those in WT plants, under water withholding drought condition (Fig. 6d). These results indicate that overexpression of *ONAC066* suppressed while suppression of *ONAC066* stimulated the accumulation of ROS during drought stress response.

### Altered expression of *ONAC066* affected the expression of drought-responsive and ROS scavenging genes

**Fig. 7:**
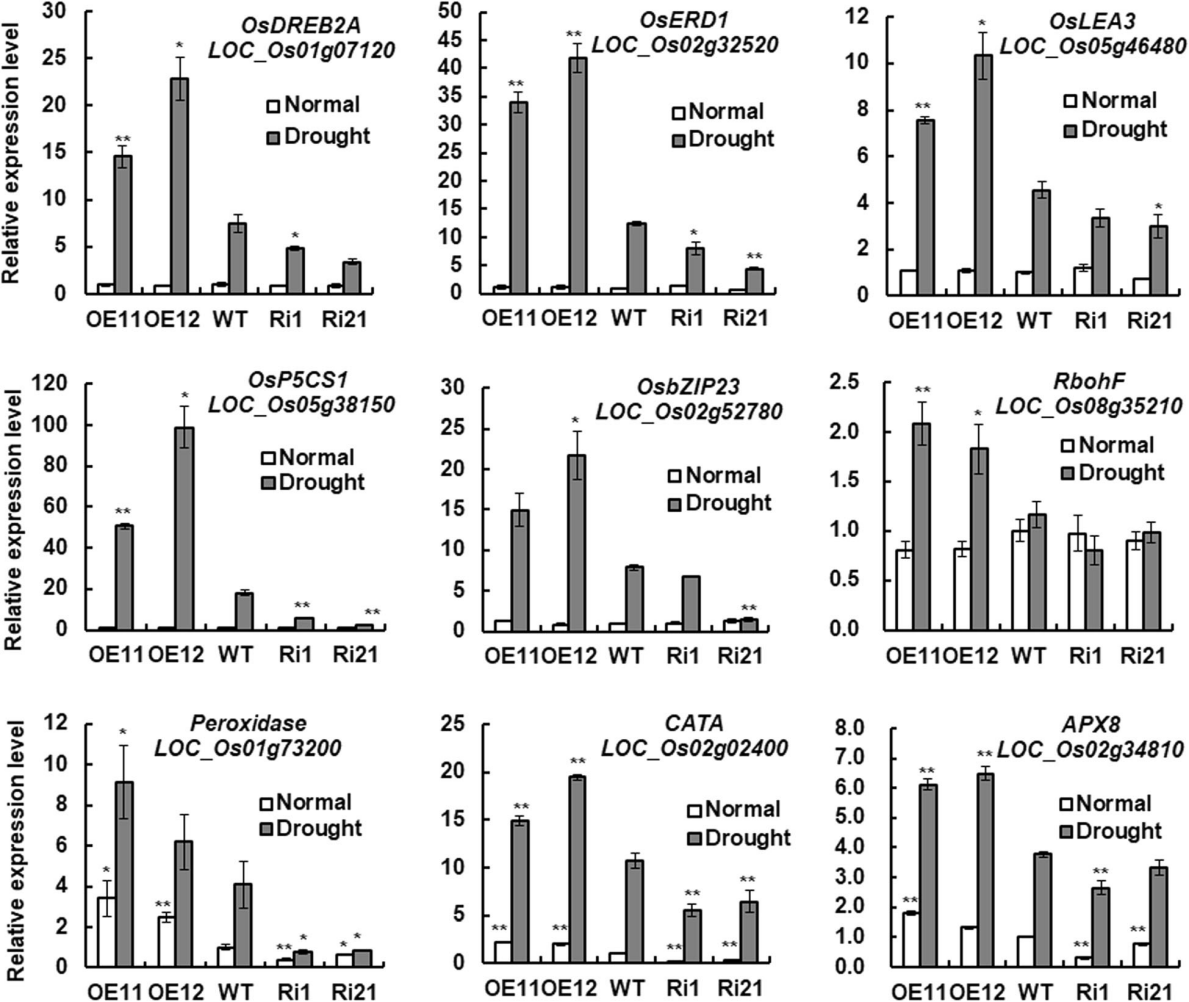
Altered expression of stress-responsive and ROS scavenging genes in ONAC066-OE and ONAC066-RNAi plants under drought condition. One group of 3-week-old ONAC066-OE, ONAC066-RNAi and WT plants were allowed for growth under normally watering condition while the other group was subjected to drought treatment by water withholding. Leaf samples were collected at 5 days after drought treatment and the expression levels of the selected genes were analyzed by qRT-PCR. Relative expression levels of the genes were normalized to the internal control of *18 s-rRNA* gene. Data presented are the means ± SD from three independent experiments and difference between ONAC066-OE/ONAC066-RNAi and WT plants are indicated by asterisks (**p < 0.01 and *p < 0.05) according to Student *t* test

To gain further insight into the possible molecular mechanism of ONAC066-meditated stress response, we analyzed and compared the expression changes of several drought-responsive genes in ONAC066-OE and ONAC066-RNAi plants with those in WT plants under normally watering and water withholding drought conditions. Under normal watering condition, the expression levels of *OsDREB2A* [55], *OsERD1* (a homolog of Arabidopsis *AtERD1* [56]), *OsLEA3* [57], *OsP5CS1* [58], and *OsbZIP23* [59] in ONAC066-OE and ONAC066-RNAi plants were comparable to those in WT plants (Fig. 7). Under water withholding drought condition, the expression levels of these drought responsive genes in ONAC066-OE and ONAC066-RNAi plants were significantly increased, as compared with those in their corresponding control plants grown under normally watering condition (Fig. 7). At 5 days after water withholding drought treatment, the expression levels of *OsDREB2A*, *OsERD1*, *OsLEA3*, *OsP5CS1* and *OsbZIP23* in ONAC066-OE lines OE11 and OE12 plants showed 1.0–2.1, 1.7–2.3, 0.7–1.3, 1.8–4.5 and 0.9–1.8 folds higher, while the expression levels in ONAC066-RNAi lines Ri1 and Ri21 plants exhibited 35–54%, 36–65%, 27–33%, 68–87% and 13–81% lower, as compared with those in WT plants, under water withholding drought condition (Fig. 7). These results indicate that overexpression of *ONAC066* upregulated while suppression of *ONAC066* downregulated the expression of drought-responsive genes during drought stress response.

We also analyzed and compared the expression changes of some ROS generating and scavenging-related genes, including *RbohF* (NADPH oxidase, LOC_Os08g35210), *POD* (peroxidase, LOC_Os01g73200), *CATA* (catalase, LOC_Os02g02400) and *APX8* (ascorbate peroxidase, LOC_Os02g34810) [32], in ONAC066-OE and ONAC066-RNAi plants with those in WT plants under normally watering and water withholding drought conditions. Expression of *RbohF* in ONAC066-OE and ONAC066-RNAi plants under normally watering condition and in ONAC066-RNAi plants under water withholding drought condition was comparable to those in WT plants; however, its expression in ONAC066-OE plants under water withholding drought condition was significantly increased, showing 1.0–1.3 folds of increase, as compared with that in WT plants (Fig. 7). Under normally watering condition, the expression levels of *POD*, *CATA* and *APX8* in ONAC066-OE lines OE11 and OE12 plants were to be 1.5–2.4, 1.2–1.0 and 0.3–0.8 folds higher, respectively, while the expression levels in ONAC066-RNAi lines Ri1 and Ri21 plants showed 39–61%, 63–84% and 23–70% lower, respectively, than those in WT plants (Fig. 7). Under water withholding drought condition, the expression levels of these ROS scavenging genes in ONAC066-OE and ONAC066-RNAi plants were significantly increased, as compared with those in their corresponding control plants grown under normally watering condition (Fig. 7). At 5 days after water withholding drought treatment, the expression levels of *POD*, *CATA* and *APX8* in ONAC066-OE lines OE11 and OE12 plants were markedly upregulated by 0.5–1.2, 0.4–0.8 and 0.6–0.7 folds, respectively, while the expression levels in ONAC066-RNAi lines Ri1 and Ri21 plants were downregulated by 78–80%, 40–49% and 13–32%, respectively, as compared with those in WT plants, under water withholding drought condition (Fig. 7). These results indicate that overexpression of *ONAC066* upregulated while suppression of *ONAC066* downregulated the expression of ROS scavenging genes in transgenic rice under normal growth condition and during drought stress response.

### ONAC066 bound to a JBSL *cis*-element in *OsDREB2A* promoter

**Fig. 8:**
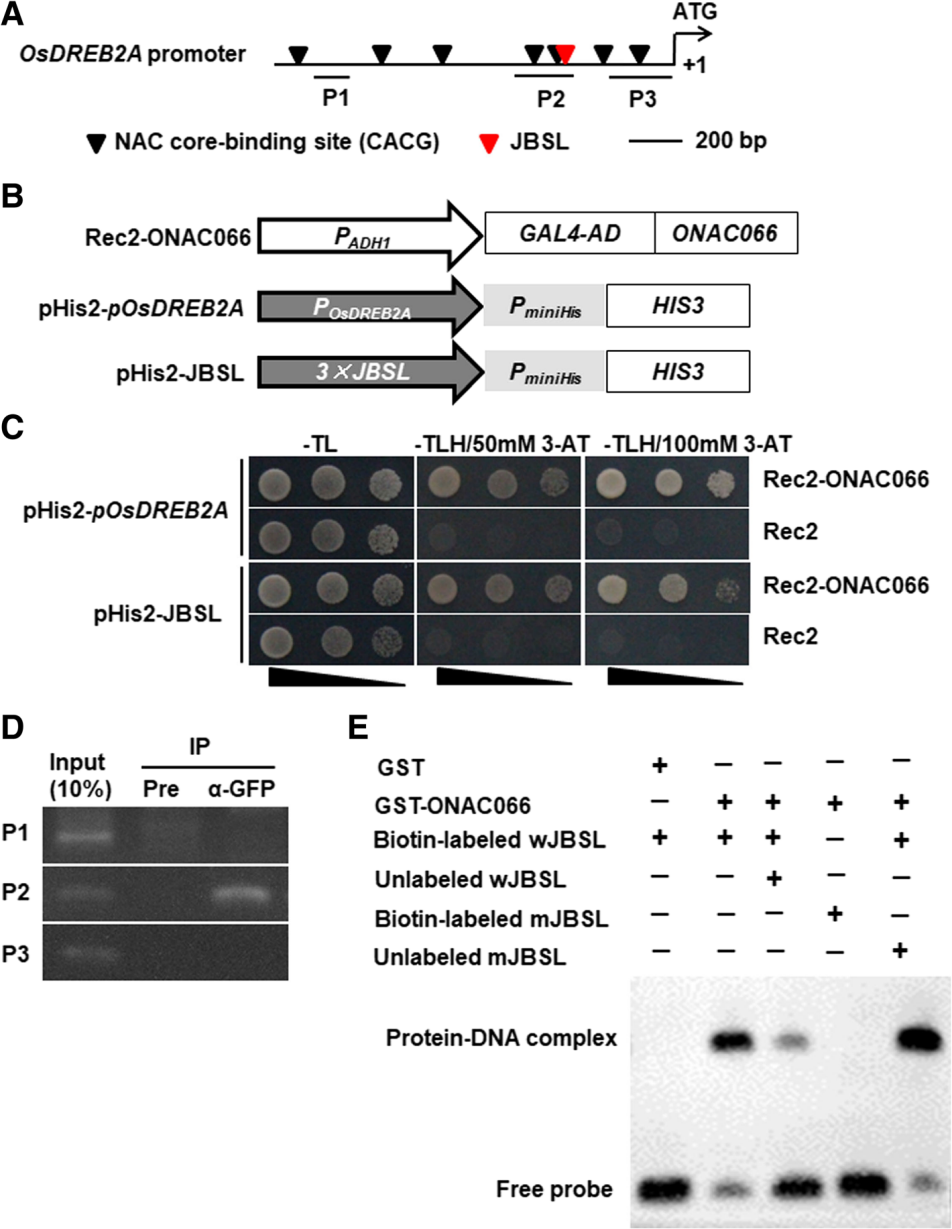
ONAC066 bound to the JBSL *cis*-element in *OsDREB2A* promoter. **a** A diagram showing putative NAC core-binding sequences and JBSL element in the promoter of *OsDREB2A*. P1, P2 and P3 were the probes used in ChIP-PCR assays. **b** Schematic diagram of the different constructs used in ONAC066-*pOsDREB2A* or ONAC066-*pOsDREB2A*-JBSL binding assays. **c** ONAC066 bound to *OsDREB2A* promoter and JBSL *cis*-element in *OsDREB2A* promoter in vivo. Rec2-ONAC066 and Rec2 empty vectors were co-transformed with the reporter vectors pHis-*pOsDREB2A* or pHis-*pOsDREB2A*-JBSL into yeast and co-transformed yeasts were dropped by a series of 10-fold dilutions on medium of SD/−Trp-Leu, SD/−Trp-Leu-His/50 mM 3-AT and SD/−Trp-Leu-His/100 mM 3-AT. Binding activity was estimated according to the growth status of the co-transformed yeasts on different medium at 2 days after plating. **d** ONAC066 bound to a JBSL-containing sequence in *OsDREB2A* promoter. ChIP of ONAC066-GFP transgenic line using GFP antibody (α-GFP) or pre-immune (Pre) serum was performed and precipitated DNA fragments were subject to PCR analysis with OsDREB2A promoter primers. 10% of chromatin amount before IP was used as positive controls (input) and IP sample with pre-immune serum was used as a negative control. **e** Binding activity of ONAC066 to JBSL *cis*-element. Biotin-labeled wJBSL and mJBSL probes or biotin-labeled wJBSL probe in combination with unlabeled wJBSL or mJBSL probe were incubated with GST-fused ONAC066 protein or a purified GST preparation as a negative control. Specific protein-DNA complexes and free probes are indicated by the arrowheads on left. Experiments in (**c**-**e**) were repeated for three times with similar results

The binding of ONAC066 to JBS element (Fig. 1c) and the changed expression of *OsDREB2A* in ONAC066-OE and ONAC066-RNAi plants during drought stress response led us to examine whether ONAC066 regulated directly the expression of *OsDREB2A* through its binding to NACARS and/or JBS elements in *OsDREB2A* promoter. Bioinformatics analysis indicated that the *OsDREB2A* promoter harbors seven typical NACRS sites and one JBSL site in 1.5 kB region upstream the ATG (Fig. 8a). The DNA binding activity of ONAC066 to the *OsDREB2A* promoter and JBSL element-containing sequence in the *OsDREB2A* promoter was examined using Y1H assays (Fig. 8b). Yeast cells co-transformed with pGADT7-Rec2-ONAC066 and pHis2-*pOsDREB2A* or pHis2–3 × JBSL grew normally while yeast cells co-transformed with pGADT7-Rec2 empty vector and pHis2-*pOsDREB2A* or pHis2–3 × JBSL failed to grow on SD/−Trp-Leu-His medium with 50 mM or 100 mM 3-AT (Fig. 8c). We further performed chromatin immunoprecipitation (ChIP) assays to determine the direct interaction between ONAC066 and the promoter of *OsDREB2A in planta*. ChIP-PCR results showed that a clear band was amplified toward the P2 probe region but no band was detected toward the P1 and P3 probe regions (Fig. 8d). These results indicate that ONAC066 bound to the P2 probe region, which contained two NACRS and one JBSL site (Fig. 8a), but not bound to P1 probe, which did not harbor NACRS site (Fig. 8a), and P3 probe, which harbored two NACRS sites (Fig. 8a), in *OsDREB2A* promoter. To confirm whether ONAC066 can bind to the JBSL *cis*-element in *OsDREB2A* promoter, recombinant GST-fused ONAC066 protein was purified and examined the binding activity using electrophoretic mobility shift assay (EMSA). The EMSA results showed that ONAC066 bound to wJBSL fragment but did not bind to the mutated fragment mJBSL (Fig. 8e). Furthermore, the binding of ONAC066 to labeled wJBSL was suppressed by excessive unlabeled wJBSL but not by excessive unlabeled mJBSL (Fig. 8e). Taken together, these results indicate that ONAC066 can bind to the JBSL *cis*-element in *OsDREB2A* promoter and thus drive the transcription of *OsDREB2A*.

## Discussion

NAC TFs have been widely implicated in regulating plant stress responses [8, 12, 18, 19]. Among the 151 members in rice NAC family [15–17], nine NAC genes, including *ONAC002*, *ONAC003*, *ONAC009*, *ONAC022*, *ONAC045*, *ONAC048*, *ONAC058*, *ONAC095* and *ONAC122*, have been shown to play roles in abiotic stress tolerance [22–34, 60]. In the present study, we demonstrated through functional analyses using overexpression and RNAi-mediated suppression transgenic rice lines that ONAC066, a rice homologue of Arabidopsis AtJUB1 [38, 39, 43], positively regulates drought and oxidative stress tolerance in rice, extending the importance of NAC TFs in plant stress response. Furthermore, biochemical studies revealed that ONAC066 regulates transcription of *OsDREB2A* through direct binding to a JBSL-containing region in its promoter.

Biochemically, most of the previously identified stress-related NAC TFs were reported to act as transcription activators, although a few of NAC TFs are transcriptional repressors [61, 62]. We found that ONAC066 has transcription activator activity and this activity is dependent on its C-terminal (Fig. 1a). This is consistent with a common knowledge that NAC TFs have a C-terminal transcriptional activation domain [22, 25, 31]. Importantly, ONAC066 can bind to a canonical NAC recognition *cis*-element fragment NACRS [14, 63] as well as to a newly identified *cis*-element JBS [39] in yeasts (Fig. 1b and c). *ONAC066* is one of the 63 stress-related *ONAC* genes with overlapping expression patterns in rice under various abiotic (e.g., drought and salt) stress conditions [20]. In this study, we verified that the expression of *ONAC066* was significantly induced by PEG6000, NaCl, H_2_O_2_ and ABA (Fig. 2). This is partially supported by the presence of several stress-related *cis*-elements including two ABREs in the promoter region of the *ONAC066* gene (Additional file 1: Figure S1C). Taken together, these observations indicate that ONAC066 is a transcription activator that can respond to multiple abiotic stress factors.

Under drought condition, the ONAC066-OE plants exhibited higher survival ratio and better growth performance while the ONAC066-RNAi plants showed opposite phenotypes (Fig. 3a). These observations demonstrate that ONAC066 is a positive regulator of drought stress tolerance in rice. This is in agreement with the functions of Arabidopsis AtJUB1, tomato SlJUB1 and banana MusaNAC042, which are homologues of ONAC066, in drought stress response [38, 39, 43–45]. The mechanism responsible for the ONAC066-regulated drought stress response can be, at least partially, explained by several physiological and biochemical changes observed in ONAC066-OE and ONAC066-RNAi plants under drought condition. First, changes in WLR and RWC in ONAC066-OE and ONAC066-RNAi plants after drought stress treatment (Fig. 3) may be one of the factors that contribute to the ONAC066-regulated drought stress response. Second, accumulation of compatible solutes such as soluble sugars and free proline is a common phenomenon in response to abiotic stress [64, 65]. We observed that ONAC066-OE plants accumulated higher levels while ONAC066-RNAi plants accumulated lower levels of free proline and soluble sugars than WT plants under drought condition (Fig. 6a and b). Similar features have been observed in transgenic rice overexpressing *ONAC009*, *ONAC058* and *ONAC022* [28, 30, 33]. Altered levels of free proline in ONAC066-OE and ONAC066-RNAi plants under drought condition may be due to the changed expression of *OsP5CS1* (Fig. 7), whose overexpression in transgenic rice led to stress-induced accumulation of free proline and increased abiotic stress tolerance [58]. Notably, the contents of free proline and soluble sugars showed an increase in ONAC066-RNAi plants after drought stress treatment, as compared to the non-stressed WT plants (Fig. 6). This imply that other mechanism(s) may compensate the function of *ONAC066* and regulate these physiological changes in ONAC066-RNAi plants upon drought stress. Third, the facts of ABA-induced *ONAC066* expression (Fig. 2d), changed expression of ABA signaling responsive stress-related genes (e.g. *OsDREB2A* [55], *OsbZIP23* [59] and *OsLEA3* [57]), and affected ABA sensitivity in ONAC066-OE and ONAC066-RNAi plants under drought condition (Figs. 5 and 7) demonstrate the involvement of ABA, as a stress hormone, in ONAC066-regulated drought tolerance. This is consistent with previous observations that overexpression of *ONAC058* [30], *ONAC002* [22], *ONAC048* [23] and *ONAC022* [33] in transgenic rice led to improved drought tolerance and hypersensitivity to exogenous ABA. However, the involvement of ABA-independent pathway in ONAC066-regulated drought tolerance cannot be ruled out as expression of *OsERD1*, which is known to be involved in ABA-independent pathway [3], was significantly affected in ONAC066-OE and ONAC066-RNAi plants (Fig. 7).

It has been shown that overexpression of Arabidopsis *AtJUB1* confers H_2_O_2_ tolerance [39]. In this study, we also observed that ONAC066-OE plants improved, while ONAC066-RNAi plants attenuated oxidative stress tolerance, as revealed by the leaf damage in H_2_O_2_ and growth performance on MV-containing medium (Fig. 4c and d). Expression ROS generating gene *RbohF* was not changed in ONAC066-OE and ONAC066-RNAi plants under normal growth condition; however, expression of ROS scavenging genes *POD*, *CATA* and *APX8* was markedly affected (Fig. 7). These observations indicate that modification of *ONAC066* expression may affect the ROS scavenging capacity, which in turn account for the changed sensitivity and tolerance to exogenous oxidative stress in ONAC066-OE and ONAC066-RNAi plants. On the other hand, ROS at physiological level is believed to act as signaling molecules that regulate plant adaptation to various stresses while ROS at excessive levels often causes significant damage to plants cells, leading to deleterious effect on stress tolerance [66, 67]. Under water withholding drought condition, relative low level of ROS was detected in ONAC066-OE plants, although expression of ROS generating gene *RbohF* and ROS scavenging genes *POD*, *CATA* and *APX8* was simultaneously upregulated (Fig. 7). By contrast, despite the unchanged expression of ROS generating gene *RbohF*, the expression of ROS scavenging genes *POD*, *CATA* and *APX8* was significantly downregulated (Fig. 7), leading to excessive accumulation of ROS, in ONAC066-RNAi plants (Fig. 6d). This is consistent with the observations that heterologous expression of *AtJUB1* in tomato lowered H_2_O_2_ level and resulted in enhanced drought tolerance while silencing of *SlJUB1* increased the accumulation of H_2_O_2_ and led to decreased drought tolerance [45]. Thus, it is likely that ONAC066 assists in regulating cellular ROS homeostasis and thereby regulates drought and oxidative stress tolerance.

It was found that AtJUB1 and SlJUB1 can directly activate transcription of *AtDREB2A* in Arabidopsis and of *SlDREB1* and *SlDREB2* in tomato, respectively [39, 45]. The promoter of *OsDREB2A*, a drought-responsive gene whose overexpression resulted in significant increase of drought stress tolerance in transgenic rice [55], harbors a JBSL *cis*-element (Fig. 8a). We further verified through Y1H, ChIP-PCR and EMSA analyses that ONAC066 binds to the JBSL *cis*-element in *OsDREB2A* promoter (Fig. 8b-e). These data, together with the expression patterns of *OsDREB2A* in ONAC066-OE and ONAC066-RNAi plants under drought stress condition (Fig. 7), imply that ONAC066 activates the expression of *OsDREB2A* through direct binding to the JBSL *cis*-element in *OsDREB2A* promoter, further demonstrating the important roles for ONAC066 in drought stress tolerance.

Modification of *ONAC066* affected the expression of many stress-responsive and ROS scavenging genes, including *OsDREB2A*, *OsERD1*, *OsbZIP23*, *OsP5CS1* and *OsAPX8*, in ONAC066-OE and ONAC066-RNAi plants during drought stress response (Fig. 7). Except that *OsDREB2A* promoter contains a JBSL element, *OsERD1*, *OsP5CS1*, *OsAPX8* and *OsbZIP23* also contain sequences similar to JBS/JBSL elements in their promoter regions (2000 bp from start codon) but the similarity is lower than 75% as compared with the characterized Arabidopsis *AtDREB2A* JBS element [39, 45] and rice *OsDREB2A* JBSL element (Fig. 8). The remaining 4 genes, including *OsLEA3*, *OsRbohF*, *POD* and *CATA*, do not contain JBS/JBSL elements in their promoters. Therefore, it is most likely that ONAC066 indirectly but not directly regulates the expression of these stress-responsive and ROS scavenging genes during drought stress response. This is partially supported by the observation that the expression levels of most of these stress-responsive and ROS scavenging genes were not affected in ONAC066-OE and ONAC066-RNAi plants under normal unstressed condition (Fig. 7). *OsDREB2A* plays critical roles in rice drought stress tolerance by acting as an upstream regulator of multiple stress response cascades [55]. The fact that ONAC066 can modulate the *OsDREB2A* expression by direct binding to the JBSL *cis*-element in its promoter may imply that OsDREB2A, acting as a master switch, links the function of ONAC066 to the expression of stress-responsive genes upon drought stress. On the other hand, the function of OsDREB2A may be regulated by numerous factors at multiple levels including ONAC066-mediated transcriptional regulations. This may partially explain the phenomena that the expression levels of these stress-responsive and ROS scavenging genes in ONAC066-RNAi plants increased after drought stress treatment, as compared with the WT levels in normal unstressed plants (Fig. 7).

## Conclusions

ONAC066 is a transcription activator that can respond to multiple abiotic stress factors. Functional analyses using overexpression and RNAi-mediated suppression transgenic rice lines demonstrate that ONAC066 acts as a positive regulator of drought and oxidative stress tolerance in rice. Furthermore, ONAC066 activates transcription of *OsDREB2A* through direct binding to the JBSL *cis*-element in *OsDREB2A* promoter. Further characterization of the ONAC066-regulated target genes at genome-wide level will provide new insights into how ONAC066 regulates the drought and oxidative stress tolerance in rice.

## Methods

### Plant materials and stress treatments

Rice cv. ZH11 (*Oryza sativa L. subsp*. *japonica*) was used in all experiments. Rice seeds (Wuhan Biorun Biotechnology Co., Wuhan, China) were sterilized in 0.3% NaClO for 15 min, germinated, and cultivated in soil mix or Yoshida’s nutrition solution in a growth room with a cycle of 16 h light at 28 °C and 8 h dark at 22 °C. For stress and ABA treatments, three-week-old seedlings were transferred to nutrients solutions containing 20% (w/v) PEG6000, 100 mM NaCl, 100 mM H_2_O_2_ or 50 μM ABA, respectively. Leaf samples were collected at indicated time points after treatment, frozen in liquid nitrogen, and stored at − 80 °C until further analysis.

### Binary vector construction, rice transformation and characterization of ONAC066 transgenic lines

The coding sequence of *ONAC066* was cloned into pCAMBIA1300s vector to fuse with GFP at C-terminal under the control of *Cauliflower mosaic virus* (CaMV) 35S promoter, yielding pCAMBIA1300s-ONAC066-GFP. A fragment of 239 bp in 3′ divergent region of *ONAC066* was amplified and cloned into RNAi vector pTCK303, yielding pTCK303-ONAC066. The constructed pCAMBIA1300s-ONAC066-GFP and pTCK303-ONAC066 were transformed into ZH11 using *Agrobacterium*-mediated method [68]. T2 generation of the transgenic lines was assessed for segregation of hygeomycin (Hgr) resistant phenotype on 1/2 MS medium containing 50 μg/L Hgr and lines with 3:1 segregation for Hgr-resistant phenotype were chosen as single-copy transgenic lines. Homozygous transgenic lines were screened, according to 100% Hgr-resistant phenotype, by sowing seeds of T3 generation of the single-copy lines on selective medium. Homozygous single-copy transgenic lines were used for all experiments. Leaf samples were collected from 3-week-old ONAC066-OE and ONAC066-RNAi plants for analyses of the expression level of *ONAC066* and the accumulation of ONAC066-GFP fusion protein. Primers used for vector construction are listed in Additional file 4: Table S1.

### Subcellular localization assays

Agrobacteria harboring pCAMBIA1300s-ONAC066-GFP and pCAMBIA1300s-GFP empty vector were separately infiltrated into leaves of 3-week-old *Nicotiana benthamiana* plants expressing a red nuclear marker RFP–H2B protein [54] using 1-mL needless syringes. GFP fluorescence was examined at 48 h after agroinfiltration and signal was excited at 488 nm and detected using a 500–530 nm emission filter under a Zeiss LSM 510 Meta confocal laser scanning microscope (Zeiss, Oberkochen, Germany).

### Y1H assays

For transcriptional activation assays, the coding sequence and the truncated sequences of *ONAC066* were obtained by PCR with different pairs of gene-specific primers (Additional file 4: Table S1) and cloned into pGBKT7 vector to fuse with GAL4 DNA binding domain. The recombinant pGBKT7 vectors, pGBKT7 empty vector (as a negative control) and pGBKT7-ONAC022 (as a positive control) [33] were transferred into yeast strain AH109, respectively. The transformed yeast cells were grown on SD/−Trp or SD/−Trp-His medium and incubated for 3 days at 30 °C, followed by addition of 5-bromo-4-chloro-3-indolyl-α-D-galactopyranoside (x-α-gal). Transactivation activity of the fused ONAC066 and its truncated mutants was evaluated according to the growth and production of blue pigments after addition of x-α-gal on SD/−Trp-His/5 mM 3-AT medium.

For DNA binding assays, the coding sequence of *ONAC066* was PCR amplified with a pair of gene-specific primers (Additional file 4: Table S1) and fused to the GAL4 activation domain in pGADT7-Rec2, yielding pGADT7-Rec2-ONAC066. Three copies of NACRS, JBS and JBSL sequences in tandems were synthesized and cloned into reporter vector pHis2, yielding pHis2–3 × NACRS, pHis2–3 × JBS and pHis2–3 × JBSL. The promoter sequence (1.0 Kb upstream from ATG) of *OsDREB2A* gene was amplified with a pair of specific primers (Additional file 4: Table S1), confirmed by sequencing and cloned into reporter vector pHis2, yielding pHis2-*pOsDREB2A*. The pGADT7-Rec2-ONAC066 and pGADT7-Rec2 empty vector were co-transformed along with one of the recombinant pHis2 constructs into yeast strain Y187. The DNA binding activity of ONAC066 was evaluated according to the growth status on SD/−Trp-Leu-His/50 mM or 100 mM 3-AT medium.

### DNA binding assays

Coding sequence of *ONAC066* was amplified using gene-specific primers (Additional file 4: Table S1), inserted into pGEX-6p-1 at *Eco*RI/*Sal*I sites, and the GST-fused ONAC066 protein was purified using BugBuster GST-Bind Purification Kit (Novagen, Darmstadt, Germany) according to the manufacturer’s protocol. A 27 bp JSBL fragment in *OsDREB2A* promoter wJBSL (5′-GGGAGATGCCGTGACAGGACGCGGTTG-3′, the core sequence underlined) and a mutated version mJBSL (5′-GGGAGAAAAAAAGACAGGAAAAAGTTG-3′, the mutated nucleotides underlined) were synthesized and labeled with biotin. EMSA was performed using LightShift Chemiluminescent EMSA Kit (Thermo Fisher Scientific, Waltham, MA, USA). Binding reactions were conducted in a total of 20 μL volume containing 5 × EMSA buffer, 2 μg recombinant ONAC066 protein or GST protein (as a negative control) and 1 μL biotin-labeled wJBSL or mJBSL probes. In the competitive reactions, excess unlabeled wJBSLor mJBSL probes was added in excess of 200 times and incubated for 20 min before addition of the labeled wJBSL probe. The reaction mixtures were separated on 6% PAGE, transferred onto nitrocellulose membranes and signals from the probes were detected according to the manufacturer’s protocol.

### Stress tolerance and ABA sensitivity assays

For drought tolerance assays, 4-week-old ONAC066-OE and WT or ONAC066-RNAi and WT seedlings (*n* = 30) were grown in the same pots with three replicates and were subject to drought stress treatment by water withholding for 7 days, followed by recovery with normally watering for another 2 days. Plants that were green and healthy young leaves after re-watering were regarded as survivals and survival ratio was calculated at 2 days after re-watering. For H_2_O_2_ tolerance assays, leaf discs from 3-week-old ONAC066-OE, ONAC066-RNAi and WT plants were incubated in 1/2 MS buffer supplemented with 0.5, 1, and 1.5% H_2_O_2_ or without H_2_O_2_ (as a control) under illumination condition at moderate light intensity (200 mmol/m^2^ s). Photographs were taken and samples were collected for analysis of chlorophyll contents at 2 days after treatment. For MV tolerance assays, germinated seeds of ONAC066-OE, ONAC066-RNAi and WT lines were planted on 1/2 MS medium supplemented with 3 μM MV or without MV (as a control) and grown for 10 days. Photographs were taken and samples were collected for analysis of chlorophyll contents at 10 days after seed sowing. For ABA sensitivity assays, 60 seeds for each of ONAC066-OE, ONAC066-RNAi and WT lines were germinated on 1/2 MS medium supplemented with 2 μM or 5 μM ABA or without ABA (as a control) in growth room with a cycle of 16 h light at 28 °C and 8 h dark at 22 °C. Photographs were taken and shoot and root length of the seedlings was recorded at 10 days after germination.

### Physiological measurements

WLR and RWC in drought stress tolerance assays were determined according to a previously reported method [69]. Briefly, fully expanded leaves of 3-week-old ONAC066-OE, ONAC066-RNAi and WT plants were detached to record the leaf fresh weight (Wf), turgid leaf weight (Wt) and dry weights (Wd), and WLR and RWC were calculated following the equations: WLR (%) = (Wf-Wt)/(Wf-Wd) × 100% and RWC(%) = (Wf − Wd)/(Wt − Wd) × 100%. Measurement of chlorophyll content in oxidative stress tolerance assays was performed as described previously [70] using 0.5 g of leaf samples and the chlorophyll content was calculated according to the formula Chl (A + B) = 5.24 × OD_664_ + 22.24 × OD_648_.

For analyses of physiological changes and gene expression under drought stress condition, one group of 3-week-old ONAC066-OE, ONAC066-RNAi and WT plants were subject to drought treatment by water withholding while the other group of seedlings were grown under normally watered condition. Leaf samples were collected at 5 days after drought treatment and used for further analyses. Contents of free proline was measured by sulphosalicylic acid method [71]. Leaf tissues (0.2 g) were extracted in 5 ml 3% sulfosalicylic acid, and boiled in water for 10 min. After cooling, 2 ml supernatant was incubated with 2 ml glacial acetic acid and 3 ml ninhydrin at 100 °C for 60 min and the reaction was terminated on ice bath. After adding 5 ml toluene and incubation at 23 °C for 24 h, the absorbance was measured at 520 nm [71]. Content of soluble sugars was determined by the anthrone method [72]. Leaf tissues (0.5 g) were extracted in 15 ml distilled water by boiling for 20 min with occasional agitation. The supernatant was filtered and brought to a final volume of 100 ml with distilled water. One milliliter of the extract was incubated with 5 mL anthrone reagent at 95 °C for 15 min, and then the reaction was terminated on ice, followed by measurement of the absorbance at 620 nm [72]. In situ detection of H_2_O_2_ and superoxide anion in leaf tissues was performed by DAB staining [73] and NBT staining [74], respectively. Quantitative measurement of H_2_O_2_ in leaf tissues was carried our using H_2_O_2_ detection kit (Nanjing Jiancheng Bioengineering Institute, Nanjing, China) following the manufacturer’s instruction.

### ChIP and PCR analysis

ChIP-PCR assays were performed as previously described [75]. Briefly, 3–4 g of 2-week-old ONAC066-OE seedlings was immediately immersed in 60 ml cross-linking buffer (0.4 M sucrose, 10 mM Tris-HCl, pH 8.0, 5 mM β-mercaptoethanol and 1% formaldehyde) under vacuum for 10 min and chromatin DNA was extracted. The chromatin DNA was sheared by sonicating into 200–500 bp fragments and pre-cleaned with salmon sperm DNA/protein A-agarose (Millipore, USA) to remove non-specific binding. The sheared DNA was incubated with GFP antibody-conjugated agarose beads (Roche, Switzerland). Agarose beads were washed and the immunoprecipitated DNA was eluted by ChIP elution buffer (1% SDS, 0.1 M NaHCO_3_). The DNA was dissociated from immunoprecipitation complex and recovered by phenol/chloroform extraction. The chromatins incubated with pre-immune (Pre) serum (GenScript, Nanjing, China) and before immunoprecipitation were used as negative controls and input controls, respectively. PCR was performed using specific primers (Additional file 4: Table S1) and the products were separated on 1.5% agarose gels and visualized by Goldview staining.

### qRT-PCR analyses of gene expression

Total RNA was extracted using RNA Isolater reagent (Vazyme Biotech, Nanjing, China) according to the manufacturer’s protocol. The remaining genomic DNA in RNA samples was removed by treating with genome DNA wiper mix (Vazyme Biotech, Nanjing, China) at 42 °C for 2 min. First-stranded cDNA was synthesized with 2 μg of purified total RNA using reverse transcription system (Vazyme Biotech, Nanjing, China). qRT-PCR was performed on a CFX96 real-time PCR system (Bio-Rad, Hercules, CA, USA) with AceQ qPCR SYBR Green Master Mix (Vazyme Biotech, Nanjing, China). Reactions were run under following conditions: 95 °C for 5 min, 40 cycles of 95 °C for 10 s, 60 °C for 15 s and 72 °C for 30 s. Rice *18 s-rRNA* gene [76] was used as an internal control to normalize data and relative expression levels of genes of interest were calculated using the 2^-ΔΔCT^ method. Gene-specific primers used in qRT-PCR are listed in Additional file 4: Table S1.

### Statistical analysis

All experiments described were performed in triplicate. Data obtained were statistically analyzed according to the Student’s *t* test and the probability value of *p < 0.05 or **p < 0.01 was considered as significant difference.

## Additional files

Additional file 1: **Figure S1.** Structural and phylogenetic features of ONAC066 and *cis*-elements in *ONAC066* promoter. **(A)** Alignment of ONAC066 with rice ONAC096, Arabidopsis AtJUB1 and tomato SlJUB1. The conserved NAC domain is underlined with red line and the five highly conserved subdomains A to E are indicated by red boxes. **(B)** Phylogenetic tree analysis of ONAC066 with other known stress-responsive rice NAC proteins in Phylogeny Group IV. Sequence alignment was performed using Clustal X1.81 program and phylogenic tree was created and visualized using MEGA 5.05. Protein sequences used for alignment are as follow: ANAC042/AtJUB1 (At2g43000), rice ONAC066 (Os03g56580), ONAC096 (Os07g04560), ONAC140 (Os12g43530), ONAC063 (Os08g33910), ONAC049 (Os08g02160), ONAC075 (Os01g66490), ONAC087 (Os05g34600), ONAC022 (Os03g04070), ONAC095 (Os06g51070), ONAC012 (Os05g37080), ONAC059/ENAC1 (Os01g64310), ONAC017/OsNAC111 (Os11g05614), ONAC134 (Os12g05990). **(C)** Distribution of major stress-related *cis*-elements in the promoter (1.5 Kb upstream of ATG) of the *ONAC066* gene. (JPG 887 kb)

Additional file 2: **Figure S2.** Tissue-specific expression of *ONAC066*. Digital expression of ONAC066 was extracted from public microarray data at NCBI (http://www.ncbi.nlm.nih.gov/geo) under accession number GSE6893). Root, 7 d seedlings; SAM, up to 0.5 mm, shoot apical meristem and rachis meristem; young inflorescence P1, up to 3 cm; P2, 3–5 cm; P3, 5–10 cm; P4, 10–15 cm, P5: 15–22 cm, P6: 22–30 cm; seeds S1, 0–2 dap; S2, 3–4 dap; S3, 5–10 dap; S4, 11–20 dap; S5, 21–29 dap. These stage specifications are estimated based on information from previous research [77]. (JPG 351 kb)

Additional file 3: **Figure S3.** Generation and characterization of ONAC066-OE and ONAC066-RNAi transgenic rice lines. **(A)** Diagrams for overexpression and RNAi constructs. **(B)** Relative expression of *ONAC066* in ONAC066-OE and ONAC066-RNAi transgenic lines. **(C)** Western blotting detection of ONAC066-GFP fusion protein in ONAC066-OE transgenic lines. (JPG 296 kb)

Additional file 4: **Table S1.** Primers used in this study. (DOCX 16 kb)

## Data Availability

The datasets supporting the results of this publication are included within the article and its Additional files.

## Abbreviations

ABA: abscisic acid
ChIP: chromatin immunoprecipitation
DAB: 3, 3′-diaminobenzidine
EMSA: electrophoretic mobility shift assay
JBS: AtJUB1 binding site
JBSL: AtJUB1 JBS-like
MS: Murashige and Skoog medium
MV: methyl viologen
NAC: NAM, ATAF and CUC
NACRS: NAC recognition sequence
NBT: nitroblue tetrazolium
OE: overexpression
qRT-PCR: quantitative reverse transcription-PCR
RNAi: RNA interference
ROS: reactive oxygen species
TF: transcription factor
WT: wild type
Y1H: yeast one hybrid
ZH11: Zhonghua11
