# Supplementary figures and images for: Rice NAC transcription factor ONAC066 functions as a positive regulator of drought and oxidative stress response
Source: BMC Plant Biol. 2019 Jun 25;19:278. doi: 10.1186/s12870-019-1883-y (PMC6593515; doi:10.1186/s12870-019-1883-y)

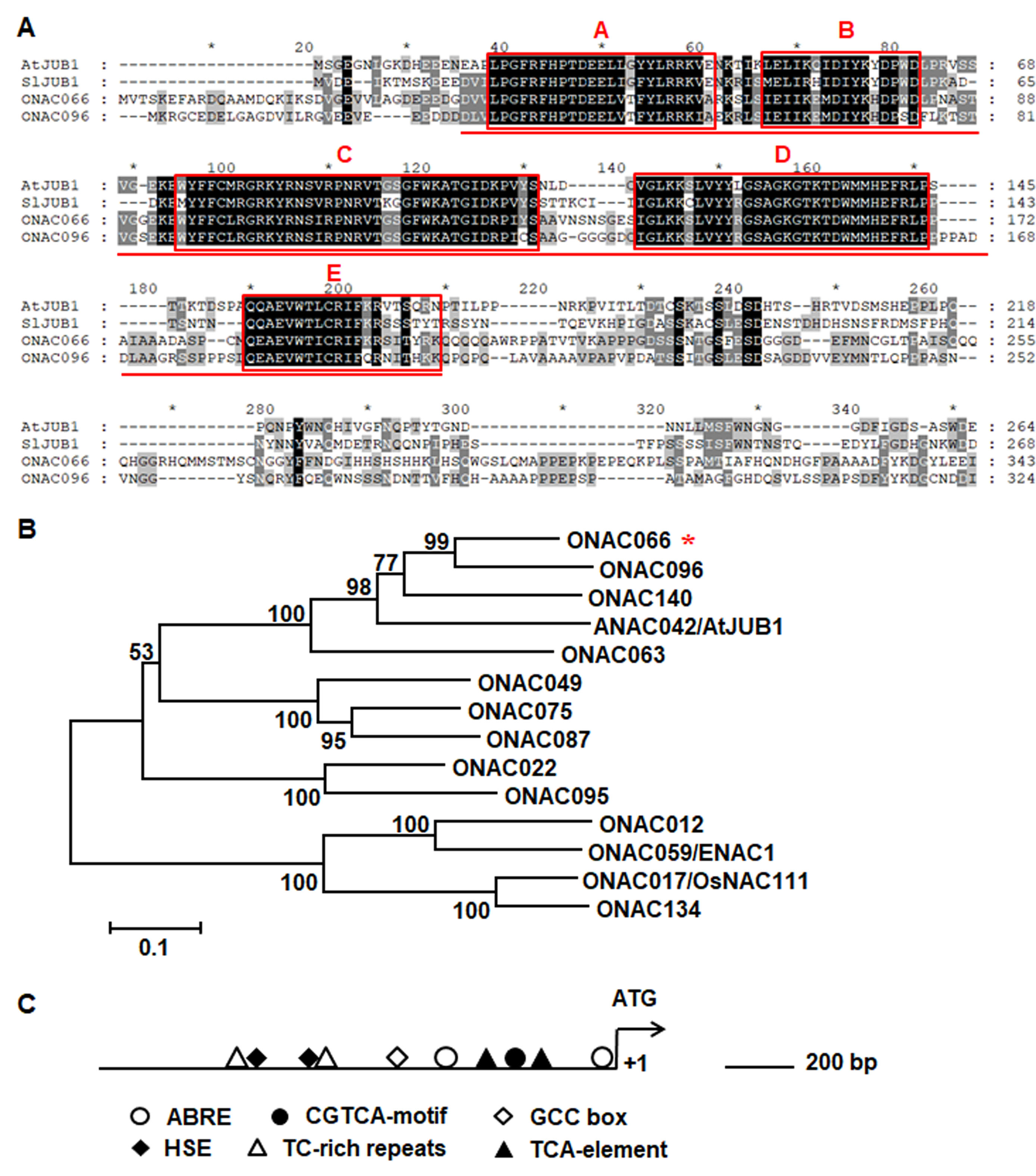

Supplement: Supplementary file 1 — Figure S1. Structural and phylogenetic features of ONAC066 and cis-elements in ONAC066 promoter. (A) Alignment of ONAC066 with rice ONAC096, Arabidopsis AtJUB1 and tomato SlJUB1. The conserved NAC domain is underlined with red line and the five highly conserved subdomains A to E are indicated by red boxes. (B) Phylogenetic tree analysis of ONAC066 with other known stress-responsive rice NAC proteins in Phylogeny Group IV. Sequence alignment was performed using Clustal X1.81 program and phylogenic tree was created and visualized using MEGA 5.05. Protein sequences used for alignment are as follow: ANAC042/AtJUB1 (At2g43000), rice ONAC066 (Os03g56580), ONAC096 (Os07g04560), ONAC140 (Os12g43530), ONAC063 (Os08g33910), ONAC049 (Os08g02160), ONAC075 (Os01g66490), ONAC087 (Os05g34600), ONAC022 (Os03g04070), ONAC095 (Os06g51070), ONAC012 (Os05g37080), ONAC059/ENAC1 (Os01g64310), ONAC017/OsNAC111 (Os11g05614), ONAC134 (Os12g05990). (C) Distribution of major stress-related cis-elements in the promoter (1.5 Kb upstream of ATG) of the ONAC066 gene. (JPG 887 kb) [file 12870_2019_1883_MOESM1_ESM.jpg]

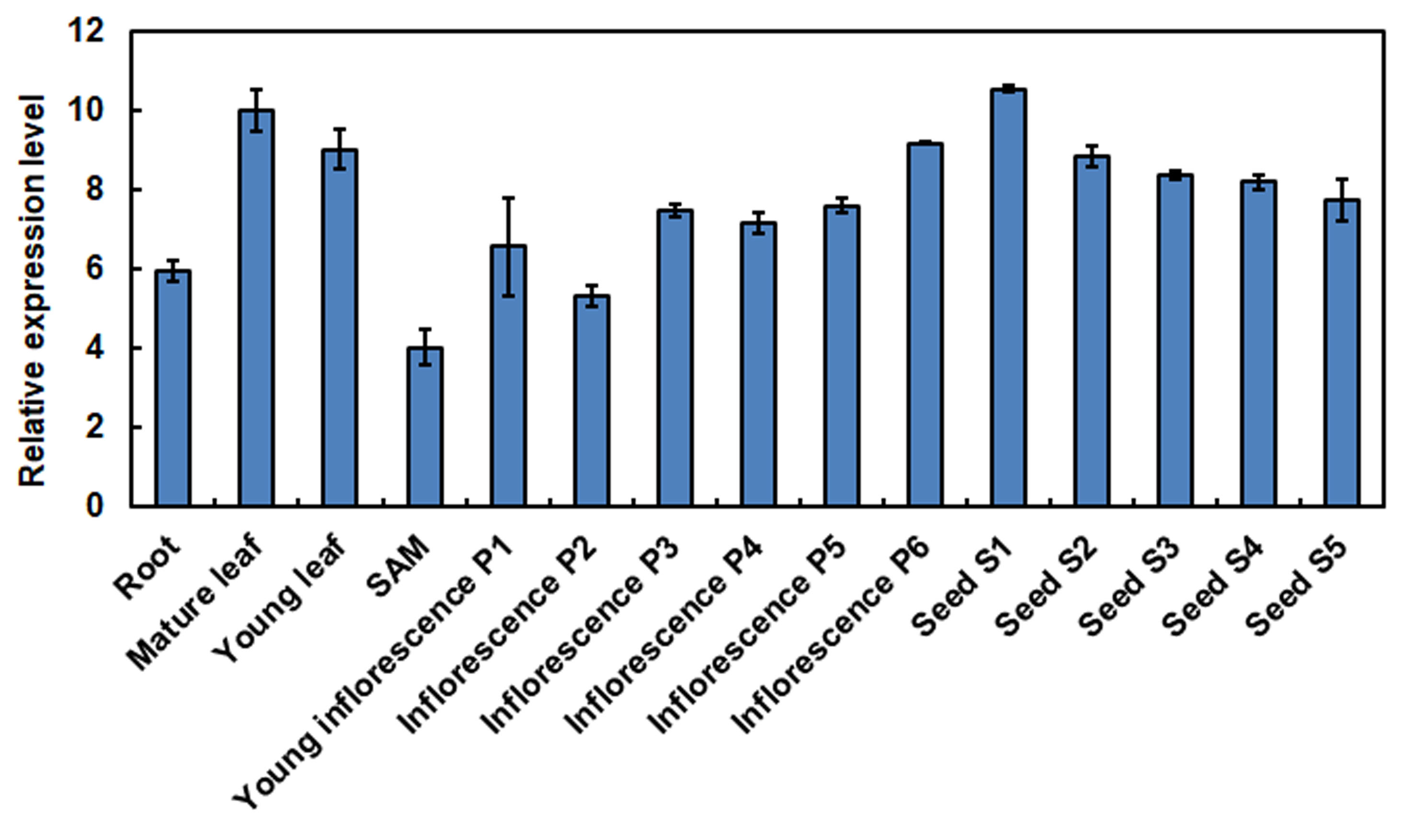

Supplement: Supplementary file 2 — Figure S2. Tissue-specific expression of ONAC066. Digital expression of ONAC066 was extracted from public microarray data at NCBI (http://www.ncbi.nlm.nih.gov/geo) under accession number GSE6893). Root, 7 d seedlings; SAM, up to 0.5 mm, shoot apical meristem and rachis meristem; young inflorescence P1, up to 3 cm; P2, 3–5 cm; P3, 5–10 cm; P4, 10–15 cm, P5: 15–22 cm, P6: 22–30 cm; seeds S1, 0–2 dap; S2, 3–4 dap; S3, 5–10 dap; S4, 11–20 dap; S5, 21–29 dap. These stage specifications are estimated based on information from previous research [77]. (JPG 351 kb) [file 12870_2019_1883_MOESM2_ESM.jpg]

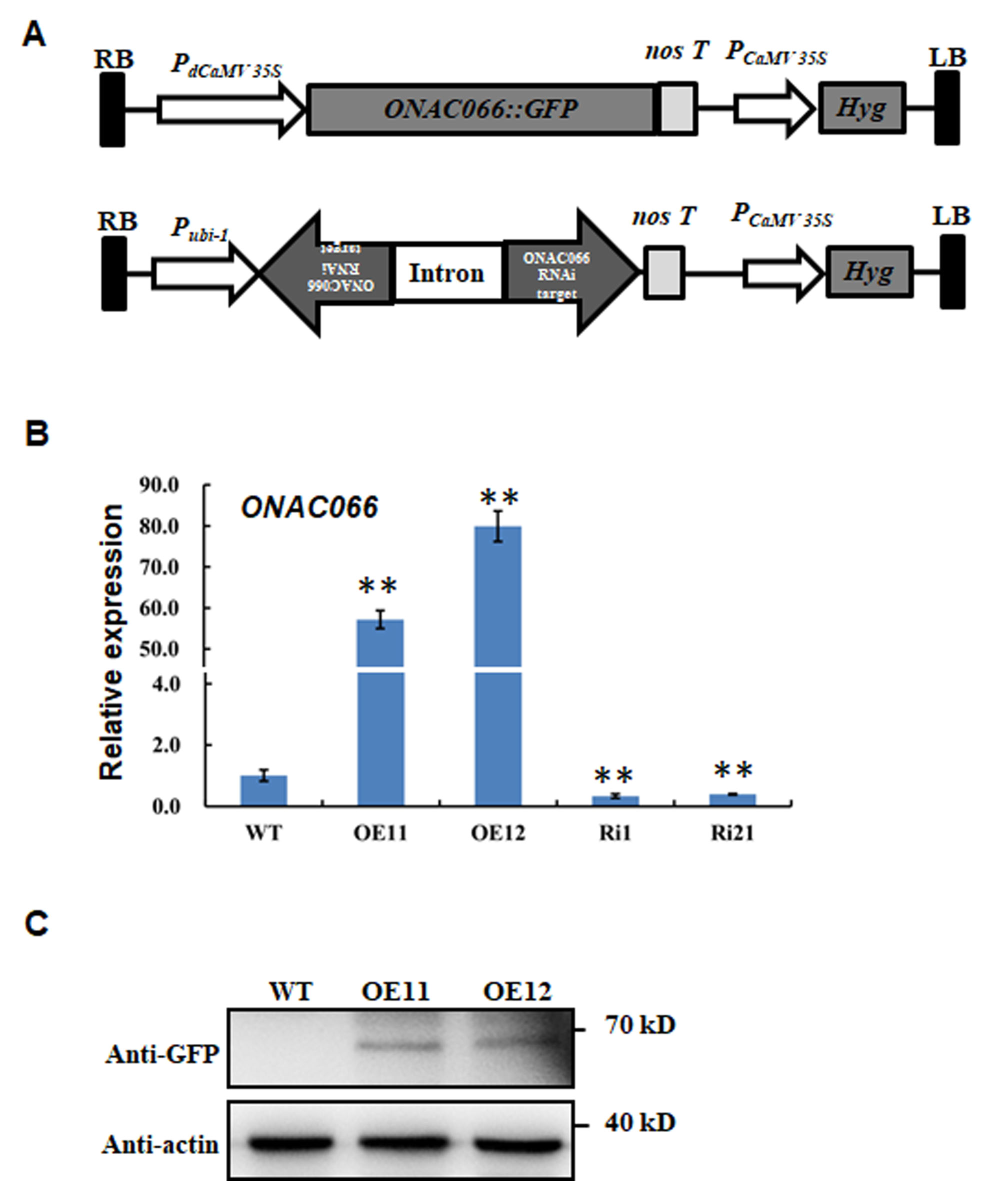

Supplement: Supplementary file 3 — Figure S3. Generation and characterization of ONAC066-OE and ONAC066-RNAi transgenic rice lines. (A) Diagrams for overexpression and RNAi constructs. (B) Relative expression of ONAC066 in ONAC066-OE and ONAC066-RNAi transgenic lines. (C) Western blotting detection of ONAC066-GFP fusion protein in ONAC066-OE transgenic lines. (JPG 296 kb) [file 12870_2019_1883_MOESM3_ESM.jpg]
