# Supplementary material for: Rice NAC transcription factor ONAC066 functions as a positive regulator of drought and oxidative stress response
Source: BMC Plant Biol. 2019 Jun 25;19:278. doi: 10.1186/s12870-019-1883-y (PMC6593515; doi:10.1186/s12870-019-1883-y)
Supplement: Supplementary file 4 — Table S1. Primers used in this study. (DOCX 16 kb) [file 12870_2019_1883_MOESM4_ESM.docx]

Additional file 3: Table S1. Primers used in this study

| Purpose | Gene | TIGR locus ID | Forward primer (5’-3’) | Reverse primer (5’-3’) |
| --- | --- | --- | --- | --- |
| Cloning | ONAC066 | LOC_Os03g56580 | ATGGTGACCAGCAAGGAGTTTG | ATATCTACAGTCATAGAATCCTGTTGGACT |
| OE construct | ONAC066 | LOC_Os03g56580 | AGCTTTCGCGAGCTCggtaccATGGTGACCAGC | GCCCTTGCTCACCATggtaccATATCTACAGTCATAGAATC |
| RNAi construct | ONAC066 | LOC_Os03g56580 | TCTGTCGACCTCGAGggtaccGCCACCACAAGCT | CAGGTCGACTCTAGAggatccTCAATATCTACAGTCATAGAATC |
|  |  |  | AGATTTTCAATCGATactagtGCCACCACAAGCTTCAT | CGATCGGGGAAATTCgagctcTCAATATCTACAGTCATAGAATC |
| Transcriptional activity construct | ONAC066 | LOC_Os03g56580 | ATGGCCATGGAGGCCgaattcATGGTGACCAGCAAGGA | CCGCTGCAGGTCGACggatccTCAATATCTACAGTCATAGAATCC |
|  |  |  | ATGGCCATGGAGGCCgaattcATGGTGACCAGCAAGGA | CCGCTGCAGGTCGACggatccGAGGCGGAACTCG |
|  |  |  | ATGGCCATGGAGGCCgaattcCCGCCGGCGATC | CCGCTGCAGGTCGACggatccTCAATATCTACAGTCATAGAATCC |
|  |  |  | ATGGCCATGGAGGCCgaattcCCGCCGGCGATC | CCGCTGCAGGTCGACggatccCATCATCTGATGACGG |
|  |  |  | ATGGCCATGGAGGCCgaattcAGCACGATGAGCTGCA | CCGCTGCAGGTCGACggatccTCAATATCTACAGTCATAGAATCC |
| Prokaryotic expression construct | ONAC066 | LOC_Os03g56580 | GgaattcCATGGTGACCAGCAAGGAG | GGgtcgacATATCTACAGTCATAGAAT |
| qPCR | 18s-rRNA | LOC_Os09g00999 | ATGGTGGTGACGGGTGAC | CAGACACTAAAGCGCCCGGTA |
|  | ONAC066 | LOC_Os03g56580 | TGCATGCAAGAAGCTGAGGTCTG | TGCTGCTTCCTGTAGGTGATGC |
|  | OsERD1 | LOC_Os02g32520 | TCAAAGGGAAGACGAAGCATGG | GGGACGGAATACAACCATCTCA |
|  | OsDREB2A | LOC_Os01g07120 | GGCTGAGATCCGTGAACCAA | GGACCATACATTGCCCTTGC |
|  | OsLEA3 | LOC_Os05g46480 | TGAAGAGCACGGTGGTCGG | GGCAGAGGTGTCCTTGTTGG |
|  | OsP5CS1 | LOC_Os05g38150 | GCTGACATGGATATGGCAAAAC | GTAAGGTCTCCATTGCATTGCA |
|  | OsbZIP23 | LOC_Os02g52780 | GGAGCAGCAAAAGAATGAGG | GGTCTTCAGCTTCACCATCC |
|  | OsPOD | LOC_Os01g73200 | AACGCAACCACCAAGCCG | CCTCGATCATGCCCATCTTGA |
|  | OsCATA | LOC_Os02g02400 | CCCCAAGGTCTCCCCTGA | AACGACTCATCACACTGGGAGAG |
|  | OsAPX8 | LOC_Os02g34810 | ATCATCGCCAGCGGATGA | GCAGCGACGAAGGGCTC |
| Y1H constructs | ONAC066 | LOC_Os03g56580 | GAGTGGCCATTATGGcccATGGTGACCAGCAA | GCCGACATGTTTTTTcccATATCTACAGTCATAGAATCC |
|  | NACRS |  | AATTCCATGTGGAGCACGGAGCACGA | CTAGTCGTGCTCCGTGCTCCACATGG |
|  | JBS |  | GAATTCGATGCCGTTAGAGACACGGATGCCGTTAGAGACACGGATGCCGTTAGAGACACGACTAGT | ACTAGTCGTGTCTCTAACGGCATCCGTGTCTCTAACGGCATCCGTGTCTCTAACGGCATCGAATTC |
|  | JBSL |  | AATTCGATGCCGTGACAGGACGCGGATGCCGTGACAGGACGCGGATGCCGTGACAGGACGCGA | CTAGTCGCGTCCTGTCACGGCATCCGCGTCCTGTCACGGCATCCGCGTCCTGTCACGGCATCG |
|  | OsDREB2A | LOC_Os01g07120 | GGgaattcTTGAAAAACGTGCATATAAA | GGactagtCGAGCGGGAACAAGAA |
| ChIP-PCR | P1 |  | ATACACATCAACGCTCAACGC | AAATAATCAAATAAACACAAAAATAGG |
|  | P2 |  | GAATGTGATATGATGTGATGAAAAG | GGGTCAACACACGAGTTGTG |
|  | P3 |  | CAAAACCCAACCCCAACCAT | CCAACTCCTCTCCCTCCCCT |
